# Exploring cost trajectories of patients admitted to short-term residential care in the Netherlands

**DOI:** 10.1371/journal.pone.0351837

**Published:** 2026-07-15

**Authors:** Eline D. Kroeze, Janet L. MacNeil Vroomen, Astrid Preitschopf, Anneke J. A. H. van Vught, Bianca M. Buurman

**Affiliations:** 1 Department of Internal Medicine, Section of Geriatric Medicine, Amsterdam UMC, Location University of Amsterdam, Amsterdam, The Netherlands; 2 Amsterdam Public Health, Aging & Later Life, Amsterdam, The Netherlands; 3 Department of Medicine for Older People, Amsterdam UMC, Location Vrije Universiteit Amsterdam, Amsterdam, The Netherlands; 4 Department of Research GRZPLUS, GRZPLUS; Omring and Zorgcirkel, Hoorn, The Netherlands; 5 Dutch Healthcare Authority (NZa), Utrecht, The Netherlands; Stanford University School of Medicine, UNITED STATES OF AMERICA

## Abstract

**Objective:**

Short-term residential care (STRC) is a Dutch form of post-acute care intended to return older adults home to live independently, yet fewer than 55% of patients are discharged home. Because post-acute care costs are unevenly distributed, average cost trajectories may obscure clinically meaningful variation. This study examines variation in STRC cost trajectories and identifies patient characteristics associated with high-cost group membership.

**Methods:**

We conducted a retrospective longitudinal observational study using national health claims data from Statistics Netherlands for patients admitted to STRC between 1 February and 31 July 2022. Reimbursed costs across seven categories (STRC, inpatient and outpatient hospital care, district care, long-term care at home, nursing home admission, and geriatric rehabilitation) were measured from one month before to five months after admission. We defined a palliative care group a priori and applied group-based trajectory modelling to the remaining cohort. Two logistic regressions assessed patient-level predictors of high-cost membership.

**Results:**

Among 16,278 patients, mean six-month costs were €29,859 (SD = €21,088). We identified an a priori palliative care group (n = 3,277; €23,200), a latent high-cost (n = 3,205; €58,478) and a latent low-cost (n = 9,796; €22,723) group. The high-cost group accounted for 39% of total costs, with the largest shares attributable to hospital care, nursing home admission, and longer STRC stays. These patients were more often readmitted to hospital within two weeks of discharge (16.9% versus 3.2%) and discharged to a nursing home (29.8% versus 10.7%). Dementia, institutional living, and several diagnosis groups (including stroke, oncology, organ failure, and cardiovascular disease) were associated with high-cost membership, but overall explanatory power was low (McFadden pseudo R² ≤ 0.05).

**Conclusions:**

STRC cost trajectories were highly skewed and poorly predicted by routinely available patient characteristics, suggesting cost variation reflects differences in care delivery more than patient case-mix. These findings point to three priorities: strengthening transitions from STRC back to home, critically evaluating STRC placement for patients likely to require nursing home admission, and scrutinizing hospital use during STRC episodes. Cost trajectories offer a promising outcome measure for evaluating intermediate and integrated care.

## Introduction

The Netherlands, like many other countries, is facing major challenges in organising sustainable healthcare for its ageing population. To manage rising demand and control expenditure, healthcare reforms were introduced in 2006 and 2015 [1,2]. Since 2015, policy has focused on ageing in place, supported by stricter admission criteria for long-term care (LTC). Nursing homes now admit only those requiring continuous care [2]. Consequently, a growing number of older adults age in place and receive care at home, delivered by professionals from various organisations operating across different healthcare sectors and funding systems [3].

As part of the 2015 reforms, the government introduced short-term residential care (STRC), a bed-based intermediate care model intended to support older adults with temporary care needs. The overarching aim is to enable patients to return home and live independently in the community. STRC targets individuals with general health problems that cannot be managed at home, but who do not require hospital admission. It serves a dual function: as step-down care for patients discharged from hospital who are not yet ready to return home, and as step-up care to avoid unnecessary hospital admissions. When needed, palliative care is provided [4]. STRC has three funding types: low-complex (support with activities of daily living (ADL) only), high-complex (includes up to 90 minutes of treatment per week), and palliative care (for patients in the last three months of life, up to 180 minutes of treatment per week) [5]. The absence of national guidelines for STRC has led to variation in how it is organised across care providers. STRC is delivered in a range of settings, including standalone STRC wards, wards co-located with geriatric rehabilitation (GR), and wards within nursing homes. These settings differ in capacity, staffing and operating practices, such as medical and multidisciplinary consultation frequency [6]. Next to STRC, there are two other bed-based intermediate care models in the Netherlands: GR and the acute geriatric community hospital (AGCH). The table in S1 Table in S1 File describes how these models differ in terms of definition, goals, admission routes and criteria, staffing and treatment.

Fewer than 55% of STRC patients returned home in 2021, which may reflect the crisis-driven nature of admissions, with patients typically presenting with multiple medical problems and palliative care needs [4]. Although national information provides basic insights, such as patient numbers, average length of stay (LOS) and discharge destinations, little is known about care use in the months following STRC discharge [7]. An observational study using 2018 data found that 33.2% of STRC patients (N = 36,660) were admitted to a nursing home within the same year. Additionally, 96.4% were hospitalised, 93.5% consulted a general practitioner, and 75.8% received home care in 2018 [6]. However, the associated costs and evoluation over time remain unclear.

Mapping cost trajectories may help to assess care use and assocated costs of STRC patients over time. Yet, analysing average trends alone may be too simplistic to inform (clinical) policy. In post-acute care, a small share of patients often accounts for a large portion of healthcare spending [8,9]. These high-cost trajectories can distort averages and mask meaningful variation across the population.

Therefore, this study aims to examine variation in STRC cost trajectories and to identify patient-level factors associated with high costs. Using claims data from Statistics Netherlands, we first desribe the average trend in STRC cost trajectories from one month before to admission to five months after admission. Next, we identify subgroups with distinct cost trajectories. Finally, we analyse whether and which patient characteristics are associated with high-cost group membership.

## Methods

### Policy context

The Dutch healthcare system is built on three principles: 1) universal access to healthcare with regulated competition among care providers, 2) solidarity via mandatory health insurance and 3) the provision of high-quality care [10]. Care for older adults is governed by three of the four national care acts (see S2 File).

### Study design

We conducted a retrospective longitudinal observational study to explore variation in cost trajectories among STRC patients from one month before admission to five months post-admission. This period captures both (pre-)admission care and recovery patterns. The chosen time window aligns with previous Dutch studies on post-acute care costs [9] and with international literature on intermediate care [11,12], where follow-up periods of 1–6 months are common. For group-based trajectory modelling (GBTM) we followed the guidelines of Nagin and Odgers [13]. For reporting, the STROBE [14] and RECORD [15] checklists were applied and are included in S3 Table in S3 File.

### Data and study population

We used nationwide claims data accessed through the secured remote environment of Statistics Netherlands (CBS). Access was granted by CBS and subject to legal and privacy restrictions. Ethical approval from a Medical Ethics Review Committee was not required, as the data contained no directly identifiable information and export of traceable data was prohibited. Data management and analysis were performed between August 1, 2024, and April 30, 2025. We included patients admitted to STRC between February 1 and July 31, 2022, to avoid overlap with 2021 (when healthcare utilization was substantially affected by COVID-19 disruptions) and to account for unavailable 2023 data. Patient characteristics were linked to claims using an anonymised population register number. Data on general practitioner care and social support were excluded due to incomplete coverage. The table in S4 Table in S4 File provides an overview of all included variables, their sources, and underlying assumptions.

### Primary outcome: trajectory costs

The trajectory costs reflected a payer perspective and included reimbursed healthcare expenditures across seven categories: STRC, district care, LTC at home, nursing home admission, GR, inpatient and outpatient hospital care. Where possible, costs were assigned to the days on which services occurred; otherwise, they were evenly distributed over the claim period. Zero-cost records were interpreted as absence of healthcare use for the corresponding service. Costs were then aggregated by month: from one month before to five months after STRC admission. All costs were adjusted for inflation using the Dutch consumer price index [16]. We also calculated costs per survival day to support interpretation of the impact of mortality within the six-month trajectory.

### Statistical analysis

We conducted four analytical steps:

1 Described average cost trajectories for the full cohort and per funding type;
2 Identified patients with palliative care funding as an a priori group and applied GBTM to the remaining cohort to identify latent cost groups;
3 Described patient and trajectories characteristics across groups;
4 Applied logistic regressions to assess patient factors associated with high-cost group membership.

Analyses were performed using Stata version 16.1. A p-value of ≤0.05 was considered statistically significant.

#### 1. Average patterns in trajectory costs

We analysed monthly and cumulative average costs from one month before to five months after STRC admission, for the full cohort and by STRC funding type, alongside monthly care utilization patterns.

#### 2. Identification of cost groups

To prevent GBTM groups from being primarily shaped by mortality patterns, patients reimbursed under the palliative STRC funding category were identified a priori as a separate cost group. GBTM was then applied to the remaining cohort to identify latent cost groups. This model-based clustering technique identifies latent patterns of temporal change solely based on structure within the data [13]. Cumulative costs were modelled across three phases (pre-admission (T1), admission (T3) and post-discharge (T5)), using a censored normal distribution, which accommodates the non-negative nature of cost data (unlike the normal distribution). Alternative distributions were not appropriate, as the outcome did not represent count (Poisson), excess-zero count (zero-inflated), or binary (logistic) data. Although gamma distributions are often preferred for right-skewed cost data, they are not currently supported within this GBTM framework; the censored normal was therefore a pragmatic alternative. Modelling cumulative cost across three phases also reduced skewness compared with monthly observations. We tested two to six trajectories and selected the optimal number using the Bayesian (BIC) and Akaike Information Criteria (AIC). Trajectory shapes were refined by varying growth terms, and the resulting groups were evaluated using four criteria: 1) average group membership probability >0.7, 2) odds of correct classification >5.0, 3) alignment between estimated and actual group sizes, and 4) acceptable confidence interval width [17,18]. We also conducted a robustness analysis restricted to patients who survived the full six-month period to assess the stability of the identified groups S8 File.

#### 3. Description of cost groups

We applied descriptive statistics to compare patient characteristics across cost groups. Variables included sex, age, migration background, living situation, income, medication use, psychotropic use, dementia diagnosis, and primary diagnosis linked to the most recent admission (hospital, GR, or ED). We also examined STRC-specific characteristics such as LOS, readmission, survival status, cost per survival day, funding type, and admission/discharge locations.

#### 4. Logistic models

Two logistic regression models were used to assess which patient-level variables were associated with high-cost group membership. Missing values for living situation and household income were recoded as an ‘unknown’ category to account for the possibility that missingness did not occur at random. Backward elimination was applied to identify the model with the lowest BIC [19]. The first model included all patients in the low- and high cost groups; the second focused on patients with a preceding admission (hospital, GR, or emergency department (ED)) to examine associations across diagnosis groups. Patients without a preceding admission or with missing primary diagnosis information were excluded (see the consort diagram in S5 Fig in S5 File).

## Results

### Sample characteristics

**Fig 1:**
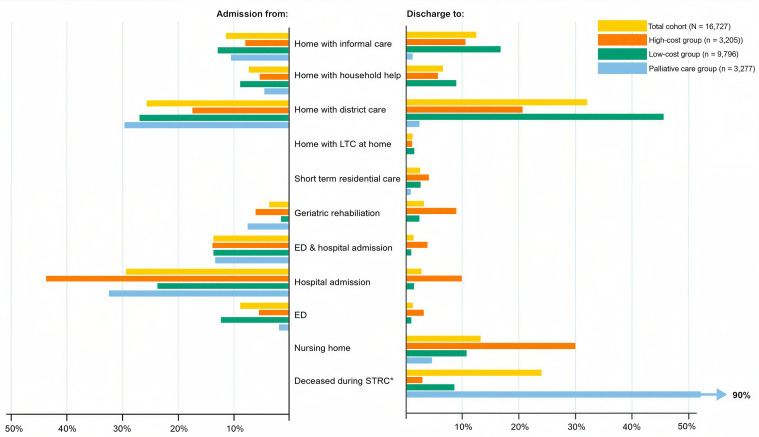
Graph representing from/ to which location patients were admitted to STRC and discharged after STRC for the total cohort and three cost groups. The yellow bars present the percentages for the total cohort (N = 16,727), the orange bars the percentages for the high-cost group (n = 3,205), the green bars the percentages for the low-cost group (9,796) and the blue bars the percentages for the palliative care group (n = 3,277). LTC = long-term care, ED = emergency department, STRC = short-term residential care. *Patients died either during STRC admission or within 14 days after discharge. Among those with palliative care funding, 88.9% died during admission and 1.6% within 14 days post-discharge, totaling 90.5%.

We included 16,278 STRC patients in the study (see consort diagram in S5 Fig in S5 File). The mean total cost per patient over the six-month trajectory was €29,859 (SD = €21,088). Baseline characteristics are summarized in Table 1. Before admission to STRC, 4% of patients had received GR, 14% had been admitted to both the ED and hospital, 29% to the hospital without ED visit, and 9% to the ED without hospitalization (see Fig 1). The main diagnoses associated with these prior admissions are provided in Table 1. Over the course of the six-month trajectory, 31% of all patients died; mortality was highest in the group with palliative care funding, with 93% dying during the trajectory.

**Table 1:** Patient characteristics for the total cohort and three cost groups.

Patient characteristics	A) Total cohort N = 16,278	B) High-cost group n = 3,205	C) Low-cost group n = 9,796	D) Palliative care n = 3,277
Male, N (%)	5,842 (36%)	1,211 (38%)	2,993 (31%)	1,638 (50%)
Age, mean (SD)	80 (11)	78 (12)	80 (11)	78 (11)
Migration background	1,188 (7%)	275 (9%)	654 (7%)	259 (8%)
Living situation before STRC admission, N (%)				
Living alone	10,725 (66%)	2,091 (65%)	6,700 (68%)	1,934 (59%)
Living together	5,287 (32%)	1,039 (32%)	2,948 (30%)	1,300 (40%)
Living in institution	208 (1%)	59 (2%)	120 (1%)	29 (1%)
Missing	58 (0%)	16 (1%)	28 (0%)	14 (0%)
Income of household in 2021, N (%)^a^				
Low income	5,341 (33%)	1,087 (34%)	3,244 (33%)	1,010 (31%)
Middle income	4,384 (27%)	882 (28%)	2,777 (28%)	725 (22%)
High income	6,428 (40%)	1,204 (38%)	3,717 (38%)	1,507 (46%)
Missing	125 (1%)	32 (1%)	58 (1%)	35 (1%)
Medication use in 2021, N (%)^b^				
0	536 (3%)	114 (4%)	297 (3%)	125 (4%)
1-4	2,866 (18%)	547 (17%)	1,825 (19%)	494 (15%)
5-9	6,124 (38%)	1,149 (36%)	3,797 (39%)	1,178 (36%)
10-14	4,561 (28%)	892 (28%)	2,682 (27%)	987 (30%)
15-19	1,711 (11%)	383 (12%)	960 (10%)	368 (11%)
>20	480 (3%)	120 (4%)	235 (2%)	125 (4%)
n ≥ 1 psychotropic drugs use in 2021, N (%)^c^	3,495 (21%)	740 (23%)	2,104 (21%)	651 (20%)
Dementia	2,582 (16%)	752 (23%)	1,606 (16%)	224 (7%)
**Primary diagnosis prior GR/ED/hospital admission**	**A) Total cohort** **n = 9,036**	**B) High-cost group** **n = 2,211**	**C) Low-cost group** **n = 5,018**	**D) Palliative care** **n = 1,807**
Stroke	261 (3%)	45 (2%)	86 (2%)	130 (7%)
Trauma	2,213 (25%)	487 (22%)	1,634 (33%)	92 (5%)
Musculoskeletal condition	1,590 (18%)	372 (17%)	1,180 (24%)	38 (2%)
Elective surgery	624 (7%)	249 (11%)	299 (6%)	76 (4%)
Oncological condition	543 (6%)	78 (4%)	61 (1%)	404 (22%)
Cardiovascular condition	479 (5%)	140 (6%)	191 (4%)	148 (8%)
Respiratory condition	255 (3%)	41 (2%)	109 (2%)	105 (6%)
Organ failure	1,300 (14%)	321 (15%)	512 (10%)	467 (26%)
Infection	705 (8%)	170 (8%)	419 (8%)	116 (6%)
Other	936 (10%)	269 (12%)	447 (9%)	220 (12%)
**Trajectory characteristics**	**A) Total cohort** **n = 16,278**	**B) High-cost group** **n = 3,205**	**C) Low-cost group** **n = 9,796**	**D) Palliative care** **n = 3,277**
Trajectory costs				
Mean trajectory costs (SD)	€29,859 (€21,088)	€58,478 (€17,651)	€22,723 (€12,267)	€23,200 (€21,380)
Median trajectory costs	€24,244	€56,364	€20,758	€16,400
LOS first admission STRC				
Mean LOS (SD)	31 (30)	44 (34)	28 (24)	25 (35)
Median LOS	22	37	22	11
Readmissions (after STRC discharge)				
≥1 STRC readmission(s)	1,353 (8%)	439 (14%)	873 (9%)	41 (1%)
≥1 ED readmission(s)	2,328 (14%)	819 (26%)	1,477 (15%)	32 (1%)
≥1 hospital admission day(s)	2,846 (17%)	1,227 (38%)	1,594 (16%)	25 (1%)
Survival				
Death during STRC admission	3,407 (21%)	34 (1%)	459 (5%)	2,914 (89%)
Death during 6-month trajectory	4,965 (31%)	452 (14%)	1,458 (15%)	3,055 (93%)
Survival days, mean (SD)	147 (58)	174 (26)	167 (41)	61 (43)
Costs per survival day (SD)	€231 (€163)	€347 (€130)	€148 (€91)	€366 (€198)
Funding type				
STRC Low complex	3,309 (20%)	392 (12%)	2,917 (30%)	0 (0%)
STRC High complex	9,692 (60%)	2,813 (88%)	6,879 (70%)	0 (0%)
STRC Palliative care	3,277 (20%)	0 (0%)	0 (0%)	3,277 (100%)

SD = standard deviation, GR = geriatric rehabilitation, ED = emergency department, LTC = long-term care, LOS = length of stay, STRC = short-term residential care.

^a^Low income is up to 140% of social minimum income, middle income is 140%−200% and high income is more than 200% of social minimum income.

^b^Based on ATC-code system. It does not include medication provided in hospitals or under the LTC- act in nursing homes.

^c^Based on ATC-codes N05A(antipsychotics), N05B(anxiolytics), N05CD (benzodiazepine), N06A(antidepressants) and N06C (antidepressants in combination with psycholeptics).

### Average pattern in trajectory costs

**Fig 2:**
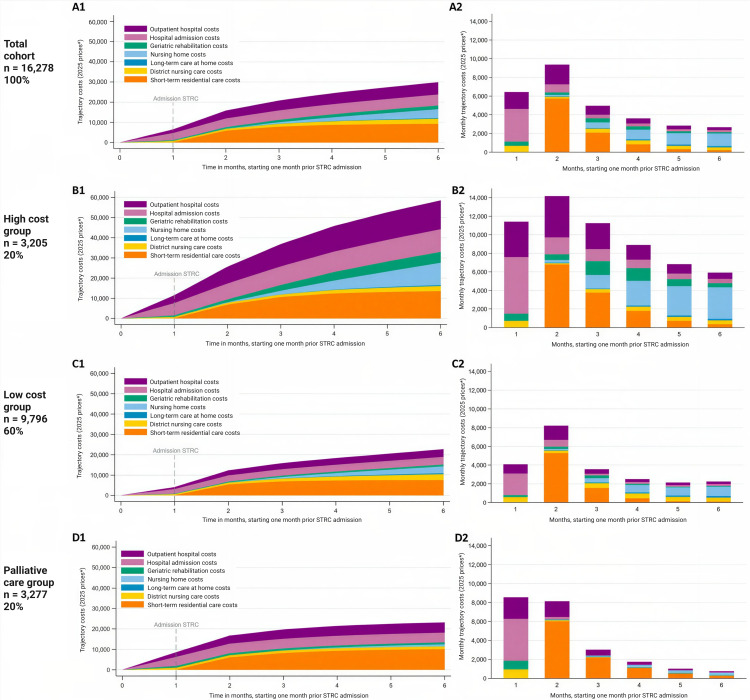
Longitudinal average patterns in trajectory costs for the total cohort and three cost groups (cumulative and monthly cost graphs). Panel A1 and A2 show the full cohort (N = 16,275), panel B1 and B2 the higher cost group (n = 3,205), panel C1 and C2 the lower cost group (n = 9,796) and panel D1 and D2 the palliative care group (n = 3,277). STRC = short-term residential stay, GR = geriatric rehabilitation, LTC = long-term care. Costs were adjusted for inflation and are presented in February 2025 prices. Table S6.1-S6.6 in S6 File present the exact costs and healthcare utilisation.

Fig 2, panel A, presents the average monthly cost trajectory across the six-month period. The cost pattern shows three distinct phases. In the pre-admission phase, costs were largely attributable to inpatient and outpatient hospital care. In the admission phase (months 1–2), STRC and outpatient hospital care were the dominant cost drivers. In the post-discharge phase (after month 3), nursing home care became the main cost driver. During the 6-month trajectory, 18% of patients were admitted to a nursing home, accounting for 14% of total trajectory costs. Full details on monthly costs and care use are included in S6 Fig and Tables in S6 File (also per STRC funding type).

### Identification of cost groups

As 93% of patients reimbursed under the palliative STRC funding category (n = 3,277) died during the 6-month trajectory (see Table 1), they were defined a priori as a separate cost group to prevent latent GBTM groups from being primarily shaped by mortality patterns. GBTM was then applied to the remaining cohort (n = 13,001). The censored normal model with two quadratic trajectories provided the best fit, based on BIC and AIC criteria in S7 Tables and Figure in S7 File.

The latent high-cost group (n = 3,205; 20%) had a mean trajectory cost of €58,478 (see Table 1). Outpatient hospital care represented 24% (€14,286) of total costs, inpatient hospital care 19% (€11,119), and nursing home care €10,999 (19%). GR accounted for €5,407 (9%), while district care contributed €2,620 (4%). LTC at home contributed minimally with €542 (1%) (see S6 File).

The latent low-cost group (n = 9,796; 60%) had a mean trajectory cost of €22,723 (see Table 1). STRC represented €7,527 (33%), outpatient hospital care €3,791 (17%), inpatient care €3,764 (17%), and nursing home care €3,186 (14%). GR contributed €999 (4%) and LTC at home €588 (3%). District care costs were relatively higher in this group (€2,869; 13%) compared to the high-cost group (€2,620; 4%) (see S6 File).

In addition to the two latent groups, panels D1 and D2 (Fig 2) presents the trajectory costs (averaging €23,200) of the a priori palliative care group (n = 3,277). This group showed high costs in month 1, were largely attributable to inpatient hospital care (€4,432, 52%), outpatient hospital care (€2,264, 26%), GR (€863, 10%) and district care (€965, 11%). Costs remained high in month 2, with STRC and hospital care (in- and outpatient) as the largest cost components. After month 2, costs declined as 89% of palliative care patients had died during STRC admission (see Table 1).

In a subgroup analysis of patients with at least six months of survival (n = 11,091), similar latent cost trajectory patterns were observed, supporting the robustness of our findings (see S8 Tables and figures in S8 File).

### Profile of cost groups

Total STRC trajectory costs amounted to €486 million (16,278 patients × €29,859). Twenty percent of patients (3,205 × €58,478) accounted for 39% of the total costs.

Compared to the lower-cost group, the high-cost group had a longer mean LOS (44 vs. 28 days) and higher rates of readmission to STRC (14% vs. 9%), the ED (26% vs. 15%), and the hospital (38% vs. 16%) during the remainder of the trajectory. Mortality during admission was lower in the high-cost group (1% vs. 5%), while six-month survival was comparable (14% vs. 15%) (see Table 1).

In the palliative care group, 89% of patients died during STRC admission, 2% within 14 days after discharge, and 3% later during the six-month trajectory. The mean survival time was 61 days. The average cost per survival day was €366; higher than in the high-cost (€347) and low-cost (€148) groups (see Table 1).

Fig 1 shows admission and discharge locations by cost group. High-cost patients were over five times more likely than low-cost patients to be admitted to the ED and/or hospital within two weeks after discharge (16.9% vs. 3.2%) and nearly three times more likely to be discharged to a nursing home (29.8% vs. 10.7%).

### Logistic regressions

Logistic regressions identified patient characteristics linked to high- versus low-cost group membership. In both models (model 1: n = 13,001; model 2: n = 7,110), backward selection excluded migration background, income, and psychotropic medication.

In model 1 (see Table S9.1. in S9 File), older age was correlated with lower odds of high-cost group membership (OR = 0.98, 95% CI: 0.98–0.98). Living in an institutional setting in 2021 was linked to 50% higher odds (OR = 1.50, 95% CI: 1.09–2.08), each additional medication type used in 2021 with 2% higher odds (OR = 1.02, 95% CI: 1.02–1.03) and dementia with 71% higher odds (OR = 1.71, 95% CI: 1.54–1.89). The model’s explanatory power was limited (McFadden pseudo R² = 0.02).

**Fig 3:**
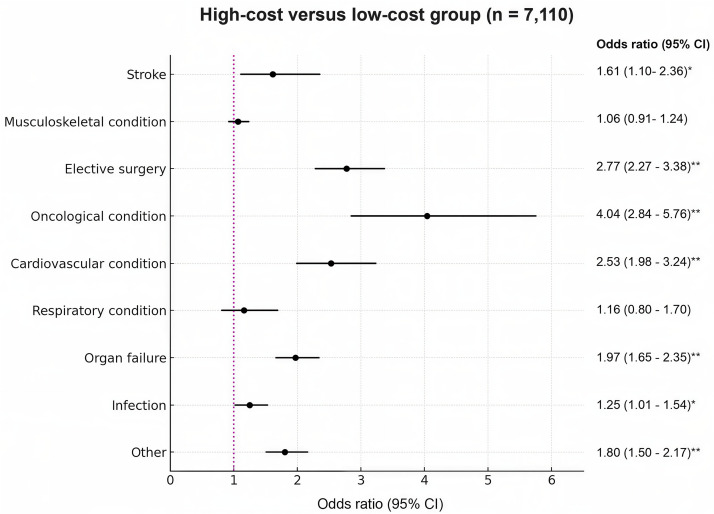
Log-scale forest plot of the odds of being in the high-cost group versus the low-cost group (Model 2). Odds ratios are shown per diagnosis group, with patients whose primary diagnosis preceding GR, ED, or hospital admission was trauma serving as the reference group. The logistic regression model was adjusted for sex, age, living situation (2021), medication count (2021), and dementia. *P  <  0.05, **P  <  0.001001.

Model 2 (see Table S9.2. in S9 File) showed diagnosis-based differences (Fig 3). Compared to trauma, higher odds for high-cost group membership were observed for stroke, elective surgery, oncological condition, cardiovascular condition, organ failure, infection and other conditions. The model’s explanatory power remained limited (McFadden pseudo R² = 0.05).

## Discussion

This study is among the first to examine cost trajectories in intermediate care and to identify patient-level factors associated with high cost trajectories. Over six months, we defined an a priori palliative care group and identified latent high- and low-cost groups. The latent high-cost group (20% of patients) accounted for 39% of total STRC trajectory costs. High costs were mainly observed in hospital care, nursing home admission, and longer STRC stays. Patient characteristics explained high-cost group membership only to a limited extent.

This is the first national study to analyse STRC cost trajectories. A key strength is the use of real-world, individual-level data covering the entire Dutch STRC population. Nonetheless, several limitations should be noted. First, the study used administrative claims data, which are not primarily collected for research purposes and capture only reimbursed care; care that was not claimed, rejected, or financed outside statutory health insurance schemes is not included. Although national coding standards apply, variation in registration practices across providers may occur, particularly for diagnosis codes. Results from logistic regression model 2 (including a categorical primary diagnosis variable) should therefore be interpreted with caution, given potential inter-provider differences in coding practices (e.g., variation in interpretation or updates from preliminary to final diagnoses). Second, clinical data reflecting somatic, functional, cognitive and social vulnerability were not available. These variables have been identified as important predictors of care use and may have improved the performance of our logistic models (assessing associations between patient characteristics with high-cost group membership) [20,21]. Third, patients with STRC palliative care funding were a priori defined as cost group and excluded from GBTM, as 93% died during the 6-month trajectory. Of the remaining cohort, 3% of the patients died during the six-month trajectory. These individuals were classified into the high-cost group if their end-of-life period was largely captured, or into the low-cost group if they died shortly after STRC admission. Although survival models have been proposed as a more appropriate approach to analyse such cost data, their application may be problematic in this context due to frequent violations of model assumptions [22,23]. Fourth, the Dutch policy and financing context limits international generalisability. Still, full population coverage and national claims data support the validity of findings within the Netherlands. Lastly, early 2022 may have been influenced by COVID-19-related disruptions (such as care delays, staffing shortages, and altered admission criteria) but it was the most recent year for which complete claims data were available.

Consistent with prior research on intermediate care, we observed a skewed distribution of healthcare expenditures, with post-discharge costs were largely attributable to hospital use and nursing home admissions [9,24]. Targeting quality improvements for high-cost patients may offer the greatest potential for enhancing access and affordability within national healthcare systems [25,26]. However, high costs should not be equated with inefficiency. As noted by Templeton et al., high expenditures can reflect necessary investments in complex, specialised, or innovative care; especially in contexts where preserving or improving quality of life is a key objective [27]. Our results also support Besselaar et al. who highlighted the heterogeneity of STRC patients [4]. This is evident in our identification of three distinct cost trajectory groups, each linked to specific patient profiles and a broad spectrum of pre-admission diagnoses. In line with previous studies, dementia was associated with high trajectory costs [28,29]. Furthermore, in line with Alridge and Kelley, we found that patients with palliative care needs incur high costs at the end of life [30].

Our findings have several clinical and policy implications. First, a substantial proportion of STRC patients were discharged to nursing homes, with this pattern again most pronounced in the high-cost group (29.8%). This raises the question for clinicians and policymakers of whether STRC is an appropriate setting for patients who are likely to require nursing home admission after discharge, given that its overarching aim is to enable patients to return home and live independently in the community. Second, a notable number of patients were readmitted to the ED and/or hospital shortly within two weeks after discharge, especially within the high-cost group (16.9%). These (unplanned) events may indicate gaps in transitional care. This may call for improvements in transitional care such as timely discharge planning, structured handovers to general practitioners, and early involvement of community nursing and social support services. Third, nearly all STRC patients received inpatient and outpatient hospital care before and after STRC admission, with particularly high use in the high-cost group. This raises questions about whether all hospital care was appropriate and the extent to which overtreatment might have been avoided through shared decision-making. Although this study does not provide evidence on the prevalence of inappropriate care, we do want to underscore the importance of clinicians and patients engaging in discussions about what constitutes potentially non-appropriate care.

Our logistic models, assessing associations between patient characteristics and high-cost group membership, showed low explanatory power, with most variance remaining unexplained. This suggests unobserved heterogeneity (e.g., somatic, functional, cognitive, and social vulnerability) that administrative claims data do not capture. For future risk-stratification in STRC, we recommend linking claims data with clinical or survey-based datasets. In addition, our retrospective longitudinal observational study design limits causal inference. Prospective cohort designs or quasi-experimental methods (such as difference-in-differences and interrupted time series) are better suited to examine causal relationships between patient characteristics and costs. Future studies could also examine whether specific interventions (such as early intensive (para)medical treatment or blended home visits) can alter cost trajectories. Moreover, multilevel analyses incorporating provider- and region-level characteristics could shed light on how differences in care delivery affect costs and other outcomes (such as LOS). Finally, this study demonstrates the value of analysing cost trajectories, which represent a valuable outcome measure for evaluating intermediate and integrated care models.

## Conclusions

STRC cost trajectories were skewed, with 20% of patients accounting for 39% of total costs. Patient characteristics explained high-cost group membership only to a limited extent. Our findings raise the question for clinicians and policymakers of whether STRC is an appropriate care setting for patients likely to require nursing home admission, and highlight priorities for strengthening transitional care from STRC to home and scrutinizing hospital use during STRC episodes. Cost trajectories represent a promising outcome measure for evaluating intermediate and integrated care

## Supporting information

S1 File Bed-based intermediate care models in the Netherlands. (PDF)

S2 File The Dutch healthcare system. (PDF)

S3 File Checklist for reporting (STROBE & RECORD). (PDF)

S4 File Variables, data sources and assumptions. (PDF)

S5 File Consort diagram. (PDF)

S6 File Average cost trajectories. (PDF)

S7 File GBTM model performance. (PDF)

S8 File GBTM robustness check. (PDF)

S9 File Results logistic linear models. (PDF)
