# Supplementary material for: Exploring cost trajectories of patients admitted to short-term residential care in the Netherlands
Source: PLoS One. 2026 Jul 15;21(7):e0351837. doi: 10.1371/journal.pone.0351837 (PMC13372163; doi:10.1371/journal.pone.0351837)
Supplement: S1 File — (PDF) [file pone.0351837.s001.pdf]

Supporting information 1

Table S1. Bed-based intermediate care models in the Netherlands. Abbreviations: ACP = Advance Care Planning; AGCH = Acute Geriatric Community Hospital; CGA = comprehensive geriatric assessment, COPD = chronic obstructive pulmonary disorder; CT = computed tomography; ED = emergency department; LTC = long-term care, MRI = magnetic resonance imaging; STRC = Short-Term Residential Care.

**Bed-based intermediate care for frail older adults in the Netherlands**		
**Geriatric revalidation**	**Short-term residential stay**	**Acute Geriatric Community Hospital**
Definition: Post-acute multidisciplinary (para)medic care for older and frail patients, including those with pre-existing functional decline or specific care needs.	*Definition:* Medical care for older adults with general health problems that do not require specialist care nor geriatric rehabilitation, but whose treatment and care needs cannot be met at home.	*Definition:* (Sub)acute specialized geriatric medical care for older patients with frailty.
Goal: To optimize functional capacities and support societal participation despite impairments, so that frail and/or older individuals can return home and live independently in the community.	*Goal:* Recovery so that older adults can return home and live independently in the community.	*Goal:* To provide medical specialist care for frail older adults in an adapted environment, so they can return home, live independently in the community and hospital (re)admissions are prevented.
Admission route:<br>Admission from hospital, ED, or home.<br>Referral by a medical specialist or ECP using a comprehensive geriatric assessment.	*Admission route:*<br>Admission from home, ED, or hospital.<br>Referral by GP or medical specialist.	*Admission route:*<br>Admission from ED.<br>Referral by a medical specialist.
Admission criteria:<br>(i). Medical stability<br>(ii). Multidisciplinary rehabilitation needs<br>(iii). Frailty and/or multimorbidity<br>(iv). Motivation/preference to undergo rehabilitation treatment<br>(v). A cognitive and physical status that allows participation in geriatric rehabilitation.<br><br>Targeted diagnoses: (1) stroke, (2) elective orthopedics, (3) trauma surgery (e.g., hip fractures), (4) amputations, and (5) other disorders (neurodegenerative diseases, oncological diseases, COPD, cardiac failure, internal- and multi-system failure).	*Admission criteria:*<br>No guidelines or targeted patient groups.	*Admission criteria (before 2023)*:<br>(i). Older patient with an acute medical problem that requires hospitalization, such as pneumonia or exacerbation of chronic conditions such as heart failure<br>(ii). Geriatric conditions (e.g. delirium, cognitive/functional impairment, falls)<br>(iii). Hemodynamic stability<br>(iv). No complex diagnostic testing needed such as CT or MRI scans during admission<br>(v). Return to previous living situation expected in 14 days
Staffing: A multidisciplinary team with special training in rehabilitation consisting of the elderly care physician, nurses, carers, physical therapists, psychologists, dieticians, social workers, and behavioral scientists.	*Staffing:* A multidisciplinary team consisting of the elderly care physician (or general practitioner), nurses, carers, physical therapists, and other paramedics if needed.	*Staffing:* An interdisciplinary team of healthcare professionals with geriatric expertise, including a geriatrician and/or medical specialist(s), elderly care physician, nurses, physical therapists, and other paramedics if needed.
Coordinating practitioner: The elderly care physician, nurse practitioner, or physician assistant.	*Coordinating practitioner:* The elderly care physician (high-complex STRC), general practitioner (low-complex STRC), nurse practitioner, or physician assistant.	*Coordinating practitioner:* The geriatrician, elderly care physician, nurse practitioner, or physician assistant.
Treatment:<br>A multidisciplinary set of evaluative, diagnostic and therapeutic interventions that are adapted to the rehabilitation needs of the frail elderly individual.<br><br>Palliative care cannot be provided in GR settings.	*Treatment:*<br>Three different STRC care paths exist:<br>- STRC low complex provides regular care for patients who are not in need of specific paramedic treatment, but temporarily need more care than homecare can provide.<br>- STRC high complex provides not only increased care, but also (multidisciplinary) treatment or rehabilitation at a slower pace than geriatric revalidation.<br>- STRC palliative care provides care for patients in the last 3 months of their life.	*Treatment:*<br>The four AGCH care components are:<br>(1) low-complex acute specialized geriatric care is safely provided<br>(2) care is patient-centered and focused on rehabilitation and return home (e.g. CGA, ACP, early rehabilitation, function focused care, caregivers involved during treatment (decisions))<br>(3) integrated care: transmural and close to home (e.g. comprehensive discharge planning (caregiver involved), warm handover to the GP and district nurse)<br>(4) fitting environment to prevent delirium and functional decline (e.g. rooming-in, noise reduction, management of delirium-inducing drugs).<br><br>Palliative care can be provided in AGCH settings.
Funding: Treatment, therapy and ADL care is funded according to a Diagnosis Treatment Combination (DTC).<br><br>--> For patients with a LTC indication, a LTC financing label ZZP 9b can be used.	*Funding:* Treatment, therapy and ADL care is funded according to a daily tariff, for a maximum of 6 months:<br>- STRC low complex: GP and paramedic treatment payment according to regular tariffs (as if patient would be home).<br>- STRC high complex: up to 1,5-hour treatment per week by ECP/paramedics.<br>- STRC hospice care: up to 3 hours treatment per week by GP/ECP/paramedics is funded.<br>--> For patients with a LTC indication, the LTC crisis financing labels (somatic or psychogeriatric) can be used.	*Funding:* Treatment, therapy and ADL care is funded according to a daily tariff, which is currently (2023) provided through an experimental financing structure.<br><br>--> For patients with a LTC indication no separate financing label has to be used under the experimental financing structure.
