# Supplementary material for: Exploring cost trajectories of patients admitted to short-term residential care in the Netherlands
Source: PLoS One. 2026 Jul 15;21(7):e0351837. doi: 10.1371/journal.pone.0351837 (PMC13372163; doi:10.1371/journal.pone.0351837)
Supplement: S2 File — (PDF) [file pone.0351837.s002.pdf]

Supporting information 2

The Dutch healthcare system

The Dutch healthcare system is broadly based on three principles: universal access to care, solidarity through mandatory medical insurance (which is provided to all), and high-quality healthcare services [1]. The system is shaped by several historical trends, changes and social conditions. The reforms in 2006 changed the role of the stakeholders and actors in the healthcare sector, while the 2015 reform was targeted towards containing costs related to long-term care [2].

Public and private insurance are merged through a universal social health insurance approach. All residents are required to purchase statutory health insurance from private insurers, who are required to accept all applicants. All insurers are mandated to operate as nonprofits. Financing is primarily public, through premiums, tax revenues, and government grants. The national government is responsible for setting health care priorities and monitoring access, quality and costs. Standard benefits include hospital, physician, home nursing, and mental health care, as well as prescription drugs. Citizens pay premiums, annual deductibles, and coinsurance or copayments on select services and drugs [3].

Three basic health care-related acts applying to care for older people

Three of the four basic health care-related acts that govern the healthcare system apply to care for older people in the Netherlands:

1. The Health Insurance Act (in Dutch: Zorgverzekeringswet) provides short-term medical care, such as general practitioner services, hospital care, prescription drugs, and mental healthcare [4]. This act accounts for the largest amount of the healthcare budget. Private insurance companies play a key role in implementing the act in a system based on “regulated competition” [1]. They must reimburse the standard benefits which are insured under the mandatory benefit package. The national government determines this statutory benefit package. Short-term medical care financed through the Health Insurance Act is provided by private entities and profits may not be distributed to the stakeholders [3].
   - ➔ Care for older people provided through the statutory benefit package includes for example hospital care, district nursing, temporary medical care for specific patient groups living at home and intermediate care (Geriatric Revalidation and Short-Term Residential Care).
2. The Long-Term Care Act (in Dutch: Wet langdurige zorg) provides long-term care for vulnerable groups such as older people with frailty, people with chronic illnesses, people with severe mental or physical disabilities. Those requiring permanent supervision and 24-hour care are entitled services under this act [1]. A special assessment center (the CIZ) determines whether a person is eligible [4]. Long-term care is covered through a state-controlled mandatory insurance and administered by regional long-term care administrators on behalf of the central government. Care is provided by private, nonprofit organizations [1].
   - ➔ The Long-Term Care Act applies to nursing at home or in a nursing home. Older people can obtain care through a contracted long-term care provider, but can also buy their own care at home through a personal budget.
3. The Social Support Act (in Dutch: Wet maatschappelijke ondersteuning) provides care to help people live independently at home and participate in society for as long as possible [1]. Social support services are financed through the municipal fund which is supplied to the (roughly 350) municipalities by the central government. The municipalities have a great deal of freedom in how they spend these funds in order to meet the requirement of the Social Support Act [3].
   - ➔ The Social Support Act provides general support to older people such as domestic help, house adjustments and transport. They can obtain care through a contracted social care provider but can also buy their own social care through a personal budget.

References:

1. Ministry of Public Health Welfare and Sport (2016) Healthcare in the Netherlands. Available from: https:// https://bit.ly/3uWLmBK
2. Kroneman M, Boerma W, van den Berg M, et al (2016) Netherlands: health system review. Word Health Organization. Available from: https://bit.ly/3RvaMhs
3. Wammes J, Stadhouders N, Westert G. International health care system profiles, Netherlands. Commonwealth Fund. Available from: https://bit.ly/3RqAH9Z
4. Varkevisser M, Schut FT, Franken F et al (2023) Sustainability and resilience in the Dutch Health System. Partnership for Health System Sustainability and Resilience (PHSSR). Available from: https://bit.ly/47ZZ0CF
