# Supplementary material for: Exploring cost trajectories of patients admitted to short-term residential care in the Netherlands
Source: PLoS One. 2026 Jul 15;21(7):e0351837. doi: 10.1371/journal.pone.0351837 (PMC13372163; doi:10.1371/journal.pone.0351837)
Supplement: S4 File — (PDF) [file pone.0351837.s004.pdf]

Supporting information 4

Table S4. Variables, data sources and assumptions.

Variable	Dataset and version used	Details and underlying assumptions
STRC costs	ZVWELV2022TABV1	Based on the claimed costs for STRC admission in 2022. Costs were equally distributed across the first and last dates of STRC admission.
Geriatric rehabilitation costs<br>Hospital admission costs<br>Outpatient hospital care costs	MSZPrestatiesVEKT2022TABV2<br>MSZZorgactiviteitenVEKT2022TABV2	Based on the claimed costs for medical specialist care in 2022. Costs were equally distributed across the days between the first and last claimed activity.<br>For hospital care, a distinction was made between hospital admission costs (defined as costs claims on days with inpatient hospitalization and/or visits to the emergency department) and outpatient costs (i.e. costs claims on days without inpatient hospitalization and/or visits to the emergency department).
District care costs	ZVWWVP2022TABV2	Based on the claimed cost for district nursing care use and costs in 2022. Costs were equally distributed across the first and last dates of the claim.
Nursing home long-term care costs<br><br>Long-term care at home costs	WLZZIN2022TABV3	Based on the claimed costs for long-term care in 2022. Costs were equally distributed across the first and last dates of the claim. Groups were formed according to the different levels of care intensity (zorgzwaartepakketten). These groups were subsequently categorized into institutional long-term care (called nursing home long-term care) and non-institutional long-term care (long-term care at home).
Sex	GBAPERSOON2022TABV2	Registered sex.
Age	GBAPERSOON2022TABV2	Age was calculated based on the date of birth and the date of admission to GR, ensuring that age reflects the moment of admission.
Migration background	GBAPERSOON2022TABV2	A dummy describing whether the individual has a first-generation migration background (i.e., the individual was not born in the Netherlands).
Living situation	GBAHUISHOUDENS2022BUSV1	The dataset includes information on an individual's living situation throughout time (longitudinal). Information on household composition on the day before GR admission was used. The categories ‘living with or without children, living alone with or without children, residing in an institution, or unknown’ were recategorized into four groups: living alone, living with others, institutionalized, and unknown.
Income	INHA2021TABV1	Registered household income in 2021 was compared to the social minimum income of that year. Low income was defined as up to 140% of the social minimum, middle income as 140%–200%, and high income as above 200% of the social minimum.
Medication use	MEDICIJNTAB2021TABV1	Medication dispensed by primary care pharmacies in 2021, based on ATC4 codes (thus excluding medication provided by hospitals and under the LTC-act). Each unique ATC4 code was considered a distinct type of medication used by the patient.
Psychotropic drug use	MEDICIJNTAB2021TABV1	A dummy variable equal to one if the individual was dispensed one or more medications in 2021 with the

		following ATC4 codes: N05A (antipsychotics), N05B (anxiolytics), N05CD (benzodiazepines), N06A (antidepressants), or N06C (antidepressant combinations).
Dementia	DEMENTIEPOPULATIEVEKTIS2022TABV1	A dummy variable equal to one if Vektis determined, based on their revalidated method, that the individual had dementia in 2022 or earlier. See https://www.vektis.nl/herijking-methode-bepaling-dementiepopulatie
Primary diagnosis	MSZPrestatiesVEKT2022TABV2<br>MSZZorgactiviteitenVEKT2022TABV2	The diagnosis of hospital, ED, or GR admission in the two weeks prior to STRC admission. The diagnosis codes from the highest-ranking care setting were used and categorized, with the ranking ordered from high to low: hospital, ED, GR admission.
Length of stay STRC	ZVWELV2022TABV1	Registered bed days were summed when consecutive, with the assumption that an interval of more than two days between stays indicates a readmission.
Readmission (STRC, ED, hospital)	ZVWELV2022TABV1<br>MSZPrestatiesVEKT2022TABV2<br>MSZZorgactiviteitenVEKT2022TABV2	Dummy variables set to one if, between STRC discharge and the end of the six-month trajectory, at least one STRC bed day, ED visit, or hospital nursing day was claimed.
Inflow<br><br>Outflow	GEBWMOTABV12022<br>ZVWWVP2022TABV2<br>WLZZIN2022TABV3<br>ZVWELV2022TABV1<br>MSZPrestatiesVEKT2022TABV2<br>MSZZorgactiviteitenVEKT2022TABV2	Categorical variables describing the highest care use in the two weeks before STRC admission (inflow) and the two weeks after STRC admission (outflow). The ranking of care use was as follows (from low to high): home without formal care (no care use), home with household help (social care use), home with district care, LTC at home, nursing home care, STRC admission, GR admission, hospital admission, ED admission, ED & hospital admission.
Survival days	GBAOVERLIJDEN2022TABV1	The number of days a person was alive during the six-month period (181.5 days) was calculated as the time between the start date and either the date of death (if occurring within the six-month trajectory) or the end of the six months.
Costs per survival day	GBAOVERLIJDEN2022TABV1	Costs per survival day were calculated by dividing the total mean costs by the number of survival days.
Data cleaning procedures		We conducted several data cleaning steps before analysis. Implausible values were identified and addressed for variables such as age, healthcare utilization, costs, and mortality. Duplicate patient identities were removed. Diagnoses were checked for internal consistency and plausibility. We also examined the dataset for duplicate cost entries. Missing data were labelled as 'unknown' and reported transparently in the descriptive tables.
Data Security Guidelines for Claims Data Processing		To prevent disclosure of participant identity, the following guidelines were applied:<br>1. Minimum number of observations: All outputs include at least 10 units (unweighted) as the basis for each cell or data point.<br>2. Models: All modelled outputs must have a minimum of 10 degrees of freedom, where degrees of freedom are calculated as the number of observations minus the number of parameters and other model constraints. Residuals and residual plots are not disclosed.

	3. Group disclosure: In all tables and similar outputs, no single cell may contain more than 90% of the total units in the respective row or column to prevent group disclosure.<br>4. Dominance: In all tables and similar data, the largest contributor to a cell must not account for more than 50% of the cell total.
