# Supplementary material for: Exploring cost trajectories of patients admitted to short-term residential care in the Netherlands
Source: PLoS One. 2026 Jul 15;21(7):e0351837. doi: 10.1371/journal.pone.0351837 (PMC13372163; doi:10.1371/journal.pone.0351837)

Supporting information 5

Fig S5. Consort diagram. STRC = Short-Term Residential Care, GBTM = group-based trajectory modelling.

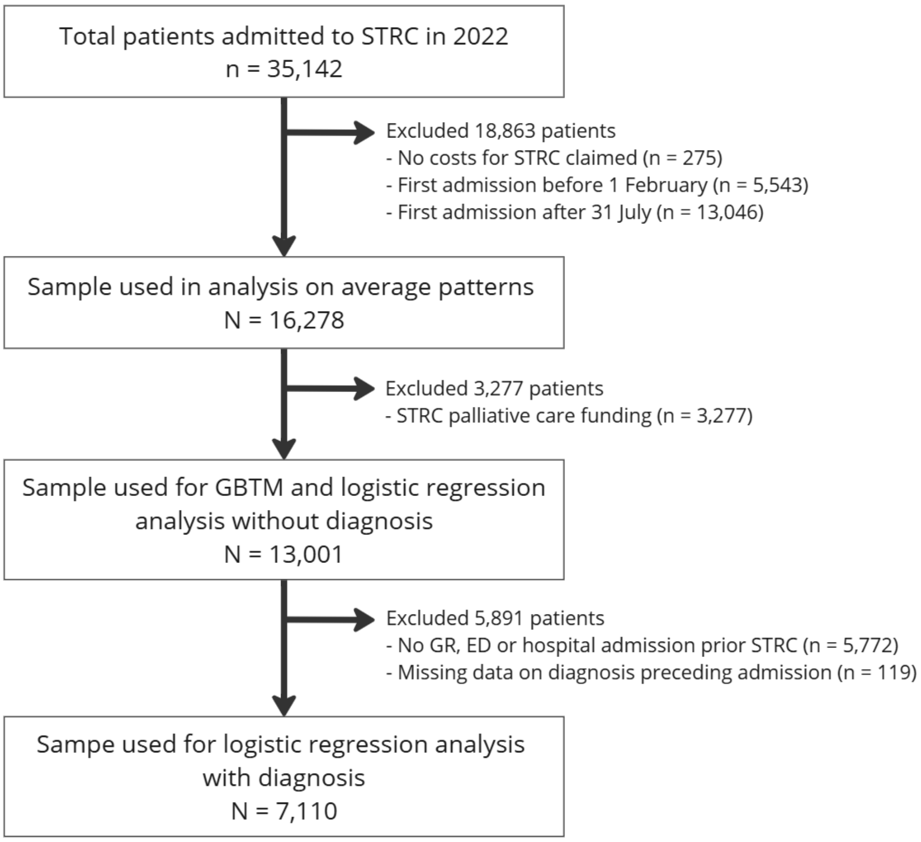

Supplement: S5 File — (PDF) [file pone.0351837.s005.pdf]
