# Supplementary material for: Exploring cost trajectories of patients admitted to short-term residential care in the Netherlands
Source: PLoS One. 2026 Jul 15;21(7):e0351837. doi: 10.1371/journal.pone.0351837 (PMC13372163; doi:10.1371/journal.pone.0351837)
Supplement: S6 File — (PDF) [file pone.0351837.s006.pdf]

Supporting information 6

Figure S6.1. Longitudinal average patterns in trajectory costs for three funding types (cumulative and monthly cost graphs). Panels A show the average trend for patients with STRC high complex funding (n = 9,692), panels B patients with STRC low complex funding (n = 3,309), panels C patients with palliative care funding (n = 3,277). STRC = short-term residential stay, GR = geriatric rehabilitation, LTC = long-term care. Costs were adjusted for inflation and are presented in February 2025 prices. Supplementary tables S5.2 -S5.4 present the exact costs and healthcare utilisation.

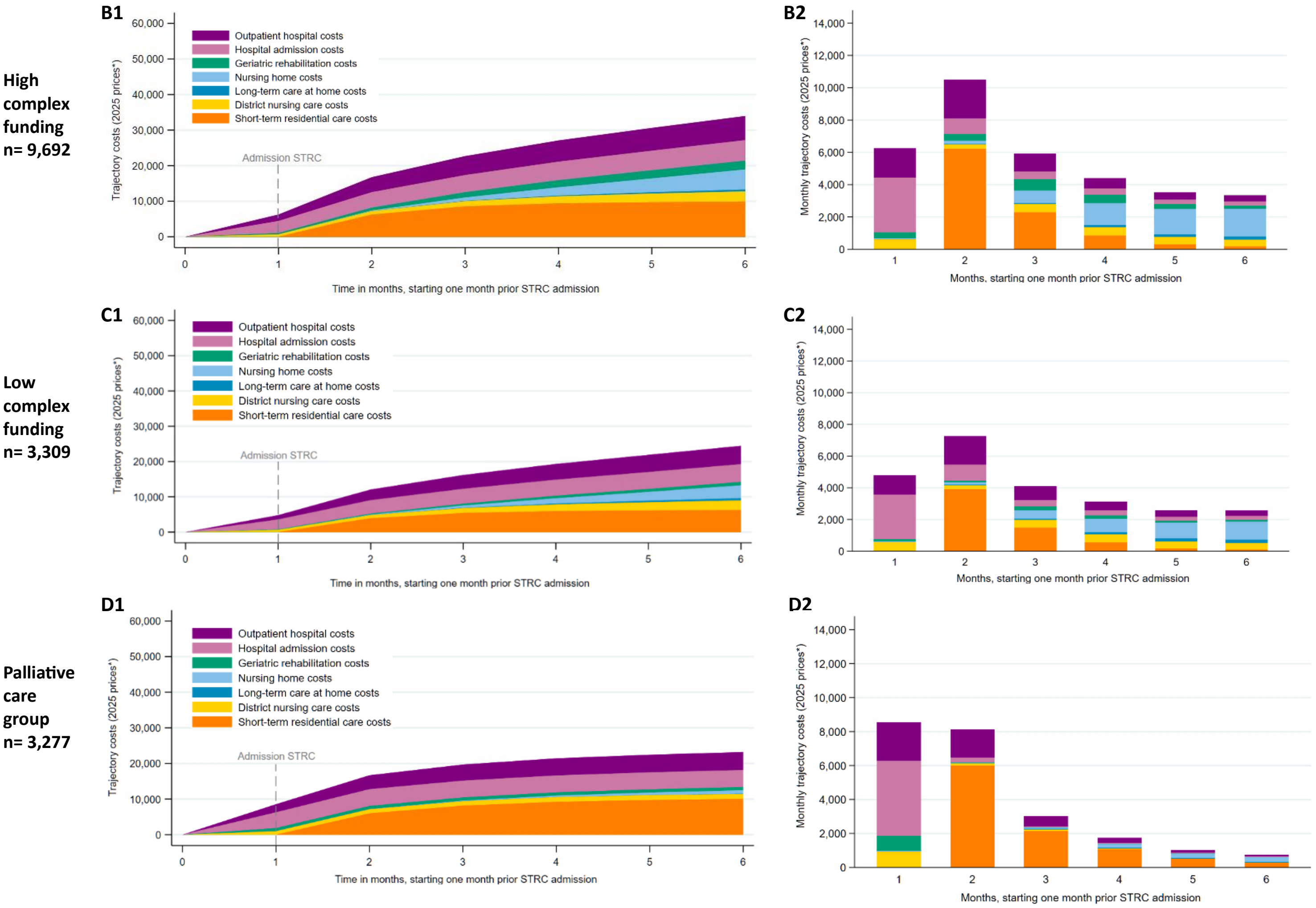

Table S6.1. Average longitudinal pattern in trajectory costs and healthcare utilization for the full STRC cohort (n = 16,272). STRC = short-term residential care, GR = geriatric rehabilitation, LTC = long-term care.

	Month 1	Month 2	Month 3	Month 4	Month 5	Month 6	Total (% of TTC)
**STRC costs (% of total monthly costs)**	€0 (0%)	€5,718 (36%)	€2,107 (38%)	€846 (36%)	€329 (33%)	€195 (30%)	**€9,194 (31%)**
**STRC use (% of patients)**	0%	100%	41%	16%	6%	3%	**100%**
**District care costs (% of total monthly costs)**	€717 (11%)	€262 (6%)	€418 (7%)	€422 (7%)	€364 (8%)	€339 (8%)	**€2,522 (8%)**
**District care use (% of patients)**	55%	43%	34%	33%	29%	27%	**73%**
**LTC at home costs (% of total monthly costs)**	€1 (0%)	€8 (0%)	€57 (0%)	€107 (1%)	€137 (1%)	€159 (2%)	**€468 (2%)**
**LTC at home use (% of patients)**	0%	1%	3%	4%	5%	5%	**7%**
**Nursing home costs (% of total monthly costs)**	€11 (0%)	€140 (1%)	€591 (4%)	€1,032 (7%)	€1,199 (11%)	€1,313 (14%)	**€4,285 (14%)**
**Nursing home use (% of patients)**	0%	4%	10%	14%	15%	15%	**18%**
**GR costs (% of total monthly costs)**	€423 (7%)	€273 (4%)	€467 (6%)	€349 (6%)	€199 (6%)	€138 (6%)	**€1,850 (6%)**
**GR use (% of patients)**	5%	6%	7%	5%	3%	2%	**14%**
**Hospital admission costs (% of total monthly costs)**	€3,482 (54%)	€843 (27%)	€369 (23%)	€305 (21%)	€225 (19%)	€210 (18%)	**€5,433 (18%)**
**Hospital admission use (% of patients)**	63%	42%	8%	7%	5%	5%	**76%**
**Outpatient care costs (% of total monthly costs)**	€1,784 (28%)	€2,121 (25%)	€960 (23%)	€550 (22%)	€381 (21%)	€313 (20%)	**€6,108 (95%)**
**Outpatient care use (% of patients)**	76%	73%	54%	45%	39%	36%	**94%**
**Total costs per category (% of TTC)**	**€6,418 (21%)**	**€9,364 (31%)**	**€4,968 (17%)**	**€3,611 (12%)**	**€2,834 (9%)**	**€2,664 (9%)**	**€29,859 (100%)**

Table S6.2. Average longitudinal pattern in trajectory costs and healthcare utilization for patients with STRC low complex funding (n = 3,309). STRC = short-term residential care, GR = geriatric rehabilitation, LTC = long-term care.

	Month 1	Month 2	Month 3	Month 4	Month 5	Month 6	Total (% of TTC)
**STRC costs (% of total monthly costs)**	€0 (0%)	€3,921 (32%)	€1,489 (33%)	€556 (31%)	€176 (28%)	€115 (26%)	**€6,258 (26%)**
**STRC use (% of patients)**	0%	100%	46%	16%	5%	3%	**100%**
**District care costs (% of total monthly costs)**	€625 (13%)	€271 (7%)	€490 (9%)	€492 (10%)	€433 (11%)	€404 (11%)	**€2,715 (11%)**
**District care use (% of patients)**	56%	51%	47%	44%	39%	35%	**78%**
**LTC at home costs (% of total monthly costs)**	€0 (0%)	€12 (0%)	€81 (1%)	€145 (1%)	€192 (2%)	€220 (3%)	**€651 (3%)**
**LTC at home use (% of patients)**	0%	1%	4%	6%	7%	7%	**10%**
**Nursing home costs (% of total monthly costs)**	€1 (0%)	€133 (1%)	€508 (4%)	€858 (8%)	€1,008 (11%)	€1,130 (15%)	**€3,638 (15%)**
**Nursing home use (% of patients)**	0%	4%	10%	13%	14%	15%	**18%**
**GR costs (% of total monthly costs)**	€144 (3%)	€115 (2%)	€246 (3%)	€211 (4%)	€115 (4%)	€116 (4%)	**€947 (4%)**
**GR use (% of patients)**	2%	3%	3%	3%	2%	2%	**7%**
**Hospital admission costs (% of total monthly costs)**	€2,807 (59%)	€1,017 (32%)	€414 (26%)	€322 (24%)	€246 (22%)	€245 (21%)	**€5,051 (21%)**
**Hospital admission use (% of patients)**	60%	41%	9%	8%	6%	6%	**75%**
**Outpatient care costs (% of total monthly costs)**	€1,216 (25%)	€1,805 (25%)	€882 (24%)	€546 (23%)	€415 (22%)	€336 (21%)	**€5,199 (21%)**
**Outpatient care use (% of patients)**	72%	79%	68%	57%	49%	47%	**97%**
**Total costs per category (% of TTC)**	**€4,792 (20%)**	**€7,274 (30%)**	**€4,112 (17%)**	**€3,130 (13%)**	**€2,584 (11%)**	**€2,567 (10%)**	**€24,459 (100%)**

Table S6.3. Average longitudinal pattern in trajectory costs and healthcare utilization for patients with STRC high complex funding (n = 9,692). STRC = short-term residential care, GR = geriatric rehabilitation, LTC = long-term care.

	Month 1	Month 2	Month 3	Month 4	Month 5	Month 6	Total (% of TTC)
**STRC costs (% of total monthly costs)**	€0 (0%)	€6,239 (0%)	€2,304 (0%)	€861 (0%)	€317 (0%)	€189 (0%)	**€9,911 (29%)**
**STRC use (% of patients)**	0%	100%	45%	16%	6%	4%	**100%**
**District care costs (% of total monthly costs)**	€664 (0%)	€289 (0%)	€496 (0%)	€518 (0%)	€452 (0%)	€420 (0%)	**€2,839 (8%)**
**District care use (% of patients)**	53%	46%	41%	40%	35%	32%	**76%**
**LTC at home costs (% of total monthly costs)**	€0 (0%)	€9 (0%)	€66 (0%)	€128 (0%)	€160 (0%)	€188 (0%)	**€551 (2%)**
**LTC at home use (% of patients)**	0%	1%	3%	5%	6%	6%	**8%**
**Nursing home costs (% of total monthly costs)**	€10 (0%)	€184 (0%)	€779 (0%)	€1,355 (0%)	€1,573 (0%)	€1,715 (0%)	**€5,615 (17%)**
**Nursing home use (% of patients)**	0%	5%	13%	17%	19%	19%	**23%**
**GR costs (% of total monthly costs)**	€370 (0%)	€413 (0%)	€967 (0%)	€512 (0%)	€292 (0%)	€190 (0%)	**€2,474 (7%)**
**GR use (% of patients)**	5%	8%	10%	8%	4%	3%	**18%**
**Hospital admission costs (% of total monthly costs)**	€3,391 (0%)	€973 (0%)	€473 (0%)	€394 (0%)	€290 (0%)	€263 (0%)	**€5,783 (17%)**
**Hospital admission use (% of patients)**	64%	45%	10%	9%	7%	6%	**80%**
**Outpatient care costs (% of total monthly costs)**	€1,816 (0%)	€2,390 (0%)	€1,106 (0%)	€638 (0%)	€450 (0%)	€381 (0%)	**€6,780 (20%)**
**Outpatient care use (% of patients)**	75%	78%	64%	54%	48%	44%	**96%**
**Total costs per category (% of TTC)**	**€6,251 (18%)**	**€10,497 (31%)**	**€5,921 (17%)**	**€4,406 (13%)**	**€3,534 (10%)**	**€3,346 (10%)**	**€33,954 (100%)**

Table S6.4. Average longitudinal pattern in trajectory costs and healthcare utilization for patients with STRC palliative care funding (n = 3,277). STRC = short-term residential care, GR = geriatric rehabilitation, LTC = long-term care.

	Month 1	Month 2	Month 3	Month 4	Month 5	Month 6	Total (% of TTC)
**STRC costs (% of total monthly costs)**	€0 (0%)	€5,989 (36%)	€2,150 (41%)	€1,092 (43%)	€518 (43%)	€291 (43%)	**€10,040 (43%)**
**STRC use (% of patients)**	0%	100%	27%	13%	6%	3%	**100%**
**District care costs (% of total monthly costs)**	€965 (11%)	€172 (7%)	€113 (6%)	€67 (6%)	€36 (6%)	€33 (6%)	**€1,387 (6%)**
**District care use (% of patients)**	59%	28%	3%	2%	2%	2%	**61%**
**LTC at home costs (% of total monthly costs)**	€3 (0%)	€2 (0%)	€5 (0%)	€9 (0%)	€10 (0%)	€8 (0%)	**€37 (2%)**
**LTC at home use (% of patients)**	0%	0%	0%	0%	0%	0%	**1%**
**Nursing home costs (% of total monthly costs)**	€24 (0%)	€17 (0%)	€117 (0%)	€251 (2%)	€288 (3%)	€308 (4%)	**€1,005 (4%)**
**Nursing home use (% of patients)**	0%	1%	2%	3%	4%	3%	**5%**
**GR costs (% of total monthly costs)**	€863 (10%)	€20 (0%)	€11 (0%)	€7 (0%)	€10 (0%)	€5 (0%)	**€916 (4%)**
**GR use (% of patients)**	11%	3%	0%	0%	0%	0%	**11%**
**Hospital admission costs (% of total monthly costs)**	€4,432 (52%)	€283 (28%)	€15 (24%)	€23 (22%)	€11 (21%)	€16 (21%)	**€4,780 (21%)**
**Hospital admission use (% of patients)**	63%	33%	1%	1%	0%	0%	**64%**
**Outpatient care costs (% of total monthly costs)**	€2,264 (26%)	€1,643 (23%)	€604 (23%)	€295 (22%)	€141 (22%)	€88 (22%)	**€5,035 (22%)**
**Outpatient care use (% of patients)**	84%	51%	11%	6%	4%	3%	**90%**
**Total costs per category (% of TTC)**	**€8,551 (37%)**	**€8,125 (35%)**	**€3,017 (13%)**	**€1,744 (8%)**	**€1,105 (5%)**	**€748 (3%)**	**€23,200 (100%)**

Table S6.5. Longitudinal average pattern in trajectory costs and healthcare utilization for the high-cost group (n = 3,205). STRC = short-term residential care, GR = geriatric rehabilitation, LTC = long-term care.

	Month 1	Month 2	Month 3	Month 4	Month 5	Month 6	Total (% of TTC)
**STRC costs (% of total monthly costs)**	€0 (0%)	€6,778 (48%)	€3,775 (34%)	€1,793 (20%)	€710 (10%)	€369 (6%)	**€13,425 (23%)**
**STRC use (% of patients)**	0%	0%	65%	32%	13%	7%	**100%**
**District care costs (% of total monthly costs)**	€747 (7%)	€188 (1%)	€370 (3%)	€467 (5%)	€437 (6%)	€410 (7%)	**€2,620 (4%)**
**District care use (% of patients)**	56%	32%	24%	29%	29%	27%	**76%**
**LTC at home costs (% of total monthly costs)**	€1 (0%)	€7 (0%)	€53 (1%)	€125 (1%)	€158 (2%)	€197 (3%)	**€542 (1%)**
**LTC at home use (% of patients)**	0%	1%	2%	3%	4%	5%	**6%**
**Nursing home costs (% of total monthly costs)**	€20 (0%)	€322 (2%)	€1,461 (13%)	€2,659 (30%)	€3,162 (46%)	€3,375 (57%)	**€10,999 (19%)**
**Nursing home use (% of patients)**	0%	8%	22%	33%	36%	37%	**41%**
**GR costs (% of total monthly costs)**	€730 (6%)	€600 (4%)	€1,495 (13%)	€1,378 (15%)	€763 (11%)	€440 (7%)	**€5,407 (9%)**
**GR use (% of patients)**	9%	12%	20%	18%	11%	6%	**32%**
**Hospital admission costs (% of total monthly costs)**	€6,110 (54%)	€1,829 (13%)	€1,305 (11%)	€908 (10%)	€606 (9%)	€440 (7%)	**€11,199 (19%)**
**Hospital admission use (% of patients)**	78%	55%	22%	17%	11%	9%	**92%**
**Outpatient care costs (% of total monthly costs)**	€3,800 (33%)	€4,444 (31%)	€2,781 (24%)	€1,586 (18%)	€988 (15%)	€686 (12%)	**€14,286 (24%)**
**Outpatient care use (% of patients)**	87%	90%	77%	64%	54%	49%	**99%**
**Total costs per category (% of TTC)**	**€11,408 (20%)**	**€14,169 (24%)**	**€11,241 (19%)**	**€8,916 (15%)**	**€6,825 (12%)**	**€5,918 (10%)**	**€58,478 (100%)**

Table S6.6. Longitudinal average pattern in trajectory costs and healthcare utilization for the low-cost group (n = 9,796). STRC = short-term residential care, GR = geriatric rehabilitation, LTC = long-term care.

	Month 1	Month 2	Month 3	Month 4	Month 5	Month 6	Total (% of TTC)
**STRC costs (% of total monthly costs)**	€0 (0%)	€5,280 (65%)	€1,547 (45%)	€453 (18%)	€141 (6%)	€105 (5%)	**€7,527 (33%)**
**STRC use (% of patients)**	0%	100%	38%	11%	3%	2%	**100%**
**District care costs (% of total monthly costs)**	€624 (15%)	€316 (4%)	€535 (20%)	€526 (34%)	€451 (34%)	€418 (29%)	**€2,869 (13%)**
**District care use (% of patients)**	53%	52%	48%	44%	39%	35%	**77%**
**LTC at home costs (% of total monthly costs)**	€0 (0%)	€10 (0%)	€75 (5%)	€135 (14%)	€172 (18%)	€196 (18%)	**€588 (3%)**
**LTC at home use (% of patients)**	0%	1%	4%	6%	7%	7%	**9%**
**Nursing home costs (% of total monthly costs)**	€3 (0%)	€122 (1%)	€464 (4%)	€760 (9%)	€862 (14%)	€974 (19%)	**€3,186 (14%)**
**Nursing home use (% of patients)**	0%	4%	9%	11%	11%	12%	**15%**
**GR costs (% of total monthly costs)**	€176 (4%)	€251 (3%)	€283 (7%)	€127 (3%)	€78 (4%)	€83 (4%)	**€999 (4%)**
**GR use (% of patients)**	2%	5%	5%	3%	1%	1%	**10%**
**Hospital admission costs (% of total monthly costs)**	€2,304 (61%)	€708 (9%)	€180 (5%)	€202 (9%)	€172 (10%)	€199 (11%)	**€3,764 (17%)**
**Hospital admission use (% of patients)**	58%	40%	6%	6%	5%	5%	**75%**
**Outpatient care costs (% of total monthly costs)**	€964 (20%)	€1,520 (18%)	€483 (14%)	€296 (14%)	€262 (15%)	€265 (14%)	**€3,791 (17%)**
**Outpatient care use (% of patients)**	70%	75%	61%	51%	46%	44%	**95%**
**Total costs per category (% of TTC)**	**€4,071 (18%)**	**€8,207 (36%)**	**€3,569 (16%)**	**€2,500 (11%)**	**€2,137 (9%)**	**€2,241 (10%)**	**€22,723 (100%)**
