# Supplementary material for: Exploring cost trajectories of patients admitted to short-term residential care in the Netherlands
Source: PLoS One. 2026 Jul 15;21(7):e0351837. doi: 10.1371/journal.pone.0351837 (PMC13372163; doi:10.1371/journal.pone.0351837)
Supplement: S7 File — (PDF) [file pone.0351837.s007.pdf]

Supporting information 7

Table S7.1 Correlation matrix total trajectory costs over time (TC_1 – TC_6) for GBTM cohort (n = 13,001).

	TC_1	TC_2	TC_3	TC_4	TC_5	TC_6
**TC_1**	1.0000					
**TC_2**	0.8317	1.0000				
**TC_3**	0.6729	0.9158	1.0000			
**TC_4**	0.5691	0.8138	0.9558	1.0000		
**TC_5**	0.4978	0.7284	0.8919	0.9742	1.0000	
**TC_6**	0.4422	0.6569	0.8297	0.9315	0.9831	1.0000

Table S7.2 BIC, AIC and group membership <5% of crude trajectory calculations with fixed quadratic growth terms to select the adequate number of groups for the GBTM cohort (n = 13,001).

Number of groups	BIC (n = 13,001)	AIC	Group membership <5%
1	-418,580.21	-418,565.27	-
2	-410,446.42	-410,416.53	0
3	-410,465.37	-410,420,53	1
4	-406,076.55	-406,016.77	1
5	-406,095.50	-406,020.77	2
6	-410,522.20	-410,432.53	3

Table S7.3 BIC, AIC and group membership <5% of crude trajectory calculations with two groups to select the adequate growth terms for the GBTM cohort (n = 13,001).

Growth terms	BIC (n = 13,001)	AIC	Group membership <5%
Linear, linear	-411,263.12	-411,240.70	0
Linear, quadratic	-410,976.68	-410,950.53	0
Quadratic, linear	-410,723.28	-411,697.13	0
Quadratic, quadratic	-410,446.42	-410,416.53	0
Quadratic, cubic	-410,451.16	-410,417.53	0
Cubic, quadratic	-410,451.16	-410,417.53	0
Cubic, cubic	-410,455.89	-410,418.53	0

Figure S7.1 Graph of GBTM results: two cubic groups with confidence intervals (n = 13,001).

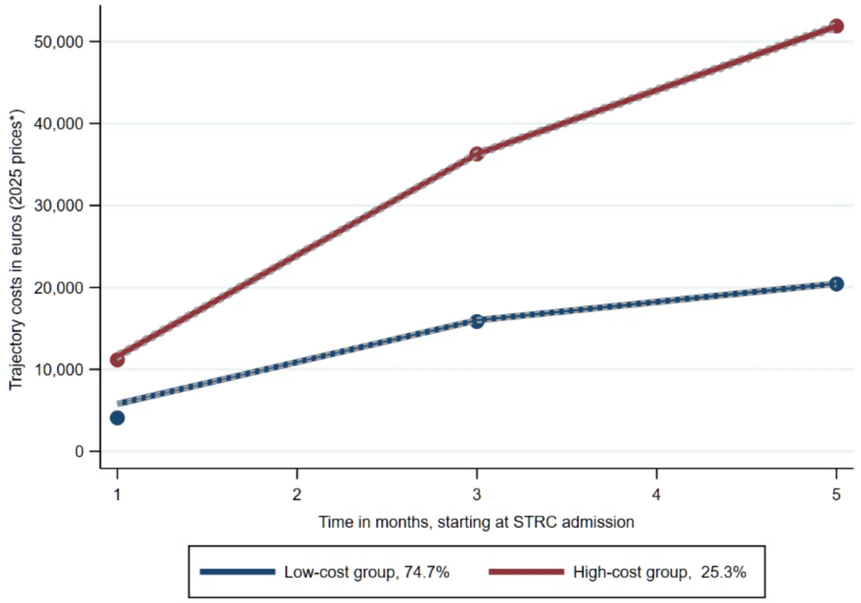

Table S7.4 Posterior diagnostics of model performance of basic trajectory model (n = 13,001).

Group	Model estimate (π^)	Proportion classified (p^)	Ave. posterior probability	Odds correct classification
1 (Higher-cost)	0.753	0.747	0.97	10.10
2 (Lower-cost)	0.247	0.253	0.93	41.23
