# Supplementary material for: Exploring cost trajectories of patients admitted to short-term residential care in the Netherlands
Source: PLoS One. 2026 Jul 15;21(7):e0351837. doi: 10.1371/journal.pone.0351837 (PMC13372163; doi:10.1371/journal.pone.0351837)
Supplement: S8 File — (PDF) [file pone.0351837.s008.pdf]

Supporting information 8

Table S8.1 Correlation matrix total trajectory costs over time (TC_1 – TC_6) for GBTM cohort of patients with at least six months of survival (n = 11,091).

	TC_1	TC_2	TC_3	TC_4	TC_5	TC_6
**TC_1**	1.0000					
**TC_2**	0.8361	1.0000				
**TC_3**	0.6800	0.9208	1.0000			
**TC_4**	0.5763	0.8200	0.9579	1.0000		
**TC_5**	0.5021	0.7325	0.8934	0.9748	1.0000	
**TC_6**	0.4451	0.6586	0.8297	0.9320	0.9832	1.0000

Table S8.2 BIC, AIC and group membership <5% of crude trajectory calculations with fixed quadratic growth terms to select the adequate number of groups for the GBTM cohort of patients with at least six months of survival (n = 11,091).

Number of groups	BIC (n = 13,001)	AIC	Group membership <5%
1	-356,343.38	-356,328.75	-
2	-349,463.66	-349,434.40	0
3	-349,482.29	-349,438.40	1
4	-349,438.40	-349,442.40	2
5	-345,783.66	-345,710.52	2
6	-345,802.28	-345,714.52	3

Table S8.3 BIC, AIC and group membership <5% of crude trajectory calculations with two groups to select the adequate growth terms for the GBTM cohort of patients with at least six months of survival (n = 11,091).

Growth terms	BIC (n = 13,001)	AIC	Group membership <5%
Linear, linear	-350,107.15	-350,085.21	0
Linear, quadratic	-349,891.90	-349,866.30	0
Quadratic, linear	-349,672.27	-349,646.67	0
Quadratic, quadratic	-349,463.66	-349,434.40	0
Quadratic, cubic	-349,468.32	-349,434.40	0
Cubic, quadratic	-349,468.32	-349,435.40	0
Cubic, cubic	-349,472.97	-349,436.40	0

Figure S8.1 Graph of GBTM results: two cubic groups quadratic groups with confidence intervals for the GBTM cohort of patients with at least six months of survival (n = 11,091).

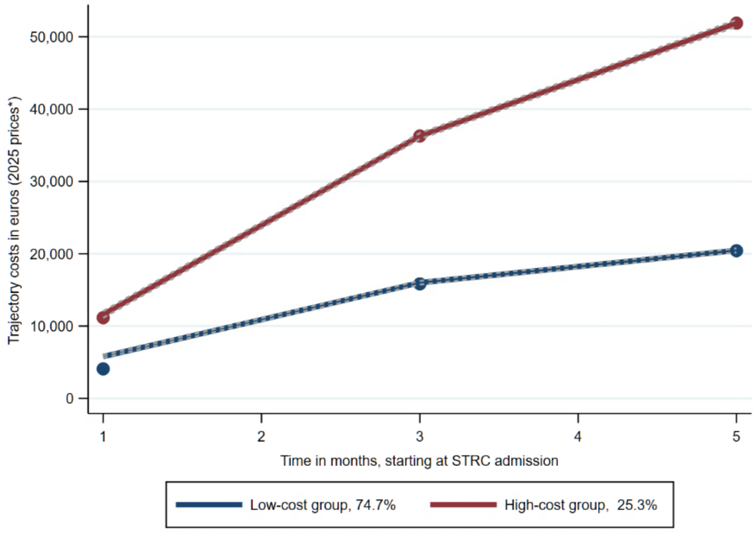

Table S8.4 Posterior diagnostics of model performance of basic trajectory model (n = 13,001).

Group	Model estimate (π^)	Proportion classified (p^)	Ave. posterior probability	Odds correct classification
1 (Higher-cost)	0.753	0.747	0.97	10.10
2 (Lower-cost)	0.247	0.253	0.93	41.23
