# Supplementary material for: Exploring cost trajectories of patients admitted to short-term residential care in the Netherlands
Source: PLoS One. 2026 Jul 15;21(7):e0351837. doi: 10.1371/journal.pone.0351837 (PMC13372163; doi:10.1371/journal.pone.0351837)
Supplement: S9 File — (PDF) [file pone.0351837.s009.pdf]

Supporting information 9

Table S9.1. Results logistic linear model for membership to the high- (versus low-) cost group (n = 13,001).

Higher cost-group	**Odds Ratio (SD)**	**95% CI**	**P>|z|**	**Low-cost group**
Male	1.29 (0.06)	1.18 - 1.40	0.000	*Reference*
Age	0.98 (0.00)	0.98 - 0.98	0.000	*Reference*
Living situation 2021				
Living alone	*Reference*	*Reference*	*Reference*	*Reference*
Living together	1.04 (0.05)	0.95 - 1.14	0.342	*Reference*
Living in an institution	1.50 (0.25)	1.09 - 2.08	0.014	*Reference*
Unknown	1.54 (0.50)	0.82 - 2.90	0.178	*Reference*
Medicine count 2021	1.02 (0.00)	1.02 - 1.03	0.000	*Reference*
Dementia	1.71 (0.09)	1.54 - 1.89	0.000	*Reference*
_cons	1.07 (0.17)	0.79 - 1.45	0.643	*Reference*
Sample-n	3,205			9,796

Table S9.2. Results logistic linear model for the subgroup of patients with GR, hospital or ED admission prior to STRC admission (n = 7,110).

**Higher vs. lower-cost group**	**Odds Ratio (SD)**	**95% CI**	**P>|z|**	**Lower cost-group**
Male	1.20 (0.07)	1.07 - 1.35	0.002	*Reference*
Age	0.98 (0.00)	0.98 - 0.99	0.000	*Reference*
Living situation 2021				
Living alone	*Reference*	*Reference*	*Reference*	*Reference*
Living together	0.87 (0.05)	0.78 - 0.98	0.021	*Reference*
Living in an institution	1.29 (0.32)	0.79 - 2.10	0.305	*Reference*
Unknown	1.17 (0.47)	0.53 - 2.57	0.697	*Reference*
Medicine count 2021	1.02 (0.00)	1.01 - 1.03	0.000	*Reference*
Dementia	1.98 (0.14)	1.71 - 2.28	0.000	*Reference*
Primary diagnosis				
Stroke	1.61 (0.31)	1.10 - 2.36	0.014	*Reference*
Trauma	*Reference*	*Reference*	*Reference*	*Reference*
Musculoskeletal condition	1.06 (0.08)	0.91 - 1.24	0.460	*Reference*
Elective surgery	2.77 (0.28)	2.27 - 3.38	0.000	*Reference*
Oncological condition	4.04 (0.73)	2.84 - 5.76	0.000	*Reference*
Cardiovascular condition	2.53 (0.32)	1.98 - 3.24	0.000	*Reference*
Respiratory condition	1.16 (0.22)	0.80 - 1.70	0.435	*Reference*
Organ failure	1.97 (0.18)	1.65 - 2.35	0.000	*Reference*
Infection	1.25 (0.13)	1.01 - 1.54	0.041	*Reference*
Other	1.80 (0.17)	1.50 - 2.17	0.000	*Reference*
_cons	0.87 (0.18)	0.59 - 1.30	0.509	*Reference*
Sample-n	2,172			4,938
