# Supplementary material for: Exploring cost trajectories of patients admitted to short-term residential care in the Netherlands
Source: PLoS One. 2026 Jul 15;21(7):e0351837. doi: 10.1371/journal.pone.0351837 (PMC13372163; doi:10.1371/journal.pone.0351837)
Supplement: Attachment — Submitted filename: Response to Reviewers PONE-D-25-54615.docx [file pone.0351837.s011.docx]

Response to Reviewers

**Manuscript Title:** Exploring cost trajectories of patients admitted to STRC in the Netherlands (PONE-D-25-54615)

Dear Academic Editor and Reviewers,

Thank you for the opportunity to revise manuscript PONE-D-25-54615. We appreciate the thoughtful comments and have addressed each point in detail, indicating the exact locations of all changes in our response to the reviewers.

A tracked and a clean version of the revised manuscript have been uploaded in accordance with the journal’s instructions. We hope the revisions improve clarity, strengthen methodological transparency, and appropriately temper the policy implications. We look forward to your reassessment.

| **Editor Comments:** | | |
| --- | --- | --- |
| Comment raised | Response by authors | Location of revisions |
| 1. Please ensure that your manuscript meets PLOS ONE's [style requirements](https://journals.plos.org/plosone/s/file?id=wjVg/PLOSOne_formatting_sample_main_body.pdf), including [those for file naming](https://journals.plos.org/plosone/s/file?id=ba62/PLOSOne_formatting_sample_title_authors_affiliations.pdf). | We have uploaded the figures as separate files (Fig1.tif/eps, Fig2.tif/eps, Fig3.tif/eps). All figures meet the required resolution of at least 300 dpi, and text within the figures is presented in Arial (8–12 pt). The maximum dimensions at 300 dpi are 2250 pixels (width) and 2625 pixels (height).  Table 1 has been revised in accordance with the table formatting guidelines. A font size of 8 was used to ensure the table fits on a single page.  The supplementary materials have been formatted according to the Guidelines for Supporting Information and uploaded as separate PDF files (S1 Files 1–7). | Fig1.tif, Fig2.tif, Fig3.tif  Table 1  S1 File 1-7 |
| 2. We note that you have indicated that there are restrictions to data sharing for this study. For studies involving human research participant data or other sensitive data, we encourage authors to share de-identified or anonymized data. However, when data cannot be publicly shared for ethical reasons, we allow authors to make their data sets available upon request. For information on unacceptable data access restrictions, please see http://journals.plos.org/plosone/s/data-availability#loc-unacceptable-data-access-restrictions.  Before we proceed with your manuscript, please address the following prompts: a) If there are ethical or legal restrictions on sharing a de-identified data set, please explain them in detail (e.g., data contain potentially identifying or sensitive patient information, data are owned by a third-party organization, etc.) and who has imposed them (e.g., a Research Ethics Committee or Institutional Review Board, etc.). Please also provide contact information for a data access committee, ethics committee, or other institutional body to which data requests may be sent.  b) If there are no restrictions, please upload the minimal anonymized data set necessary to replicate your study findings to a stable, public repository and provide us with the relevant URLs, DOIs, or accession numbers. Please see http://www.bmj.com/content/340/bmj.c181.long for guidelines on how to de-identify and prepare clinical data for publication. For a list of recommended repositories, please see https://journals.plos.org/plosone/s/recommended-repositories. You also have the option of uploading the data as Supporting Information files, but we would recommend depositing data directly to a data repository if possible.  c) In the online submission form, you indicated that *the raw data used in this study are available through the Statistics Netherlands (CBS) remote access environment for authorized researchers (CBS microdata). Access requires approval from CBS and is subject to legal and privacy restrictions. The data management procedures and programming code used for this study are available upon reasonable request from the first author*. All PLOS journals now require all data underlying the findings described in their manuscript to be freely available to other researchers, either 1. In a public repository, 2. Within the manuscript itself, or 3. Uploaded as supplementary information. This policy applies to all data except where public deposition would breach compliance with the protocol approved by your research ethics board. If your data cannot be made publicly available for ethical or legal reasons (e.g., public availability would compromise patient privacy), please explain your reasons on resubmission and your exemption request will be escalated for approval.  d) Please update your Data Availability statement in the submission form accordingly. | Thank you for your request and address prompts a, b, c and d:  a) The data used in this study cannot be made publicly available due to legal and ethical restrictions. The analyses are based on administrative claims data from Statistics Netherlands (CBS) (CBS microdata), which contain sensitive, potentially identifiable personal data. Public sharing would violate the General Data Protection Regulation (EU). In addition, the data are not owned by the research team but by CBS, and access is restricted to their secure remote environment. Qualified researchers may request access via CBS ([microdata@cbs.nl](mailto:microdata@cbs.nl)).  b) Not applicable.  c) We request an exemption from the public data deposition requirement (see our response to point a above). Public availability would compromise patient privacy and violate the General Data Protection Regulation (EU). In addition, the data are not owned by the research team. Access for other researchers is possible via CBS, but requires approval and is subject to legal and privacy restrictions (microdata@cbs.nl).  d) Not applicable | X  X  X  X  X |
| 4. Thank you for stating the following financial disclosure: *This work was supported by ZonMW, the Netherlands Organization for Health Research and Development (grant number: 10020032210003).* Please state what role the funders took in the study. If the funders had no role, please state: "The funders had no role in study design, data collection and analysis, decision to publish, or preparation of the manuscript."  If this statement is not correct you must amend it as needed. Please include this amended Role of Funder statement in your cover letter; we will change the online submission form on your behalf. | Thank you for this request. The statement provided is correct for our study. We confirm that the funder, ZonMW, had no role in the study design, data collection and analysis, decision to publish, or preparation of the manuscript. We have amended the Role of Funder statement accordingly in the Cover Letter and the Title Page. | Cover letter  Title page |
| 5. Please amend your authorship list in your manuscript file to include author line Kroeze, | We are not entirely sure we understand this comment. The title page already included the author line for Kroeze. We did not add this author line to the manuscript file to preserve anonymity for reassessment. Please let us know if you intended the title page to be included in the manuscript file, thereby making it non-anonymous; we can revise this accordingly. | Title page |
| 6. Please amend the manuscript submission data (via Edit Submission) to include author Eline D. Kroeze, | We double-checked and saw that the manuscript submission data (via Edit Submission) already included the author Eline D. Kroeze. | Manuscript submission data |
| 7. Please amend your list of authors on the manuscript to ensure that each author is linked to an affiliation. Authors’ affiliations should reflect the institution where the work was done (if authors moved subsequently, you can also list the new affiliation stating “current affiliation:….” as necessary). | All authors were already correctly linked to the most current institutions. | Title page  Manuscript submission data |
| 8. We note that there is identifying data in the Supporting Information file < Supporting material.docx>. Due to the inclusion of these potentially identifying data, we have removed this file from your file inventory. Prior to sharing human research participant data, authors should consult with an ethics committee to ensure data are shared in accordance with participant consent and all applicable local laws. Data sharing should never compromise participant privacy. It is therefore not appropriate to publicly share personally identifiable data on human research participants. The following are examples of data that should not be shared:  -Name, initials, physical address -Ages more specific than whole numbers -Internet protocol (IP) address -Specific dates (birth dates, death dates, examination dates, etc.) -Contact information such as phone number or email address -Location data -ID numbers that seem specific (long numbers, include initials, titled “Hospital ID”) rather than random (small numbers in numerical order)  Data that are not directly identifying may also be inappropriate to share, as in combination they can become identifying. For example, data collected from a small group of participants, vulnerable populations, or private groups should not be shared if they involve indirect identifiers (such as sex, ethnicity, location, etc.) that may risk the identification of study participants.  Additional guidance on preparing raw data for publication can be found in our Data Policy (https://journals.plos.org/plosone/s/data-availability#loc-human-research-participant-data-and-other-sensitive-data) and in the following article: http://www.bmj.com/content/340/bmj.c181.long.  Please remove or anonymize all personal information (<specific identifying information in file to be removed>), ensure that the data shared are in accordance with participant consent, and re-upload a fully anonymized data set. Please note that spreadsheet columns with personal information must be removed and not hidden as all hidden columns will appear in the published file. | Thank you for your careful review of the Supporting Information file. We have re-examined the file and did not identify any directly or indirectly identifying data (e.g., names, dates, locations, or unique identifiers). As far as we know, we are compliant with all ethics committee guidelines. Could you please specify which information should be removed?  If any concern remains, it may relate to the S6 Tables and Figures, which present aggregated data from subgroups. However, these outputs were formally reviewed by (privacy officers at) CBS prior export from the CBS secure environment. CBS confirmed that the data meets the disclosure control standards and that there is no risk of identifying individual participants. | X |
| 9. If the reviewer comments include a recommendation to cite specific previously published works, please review and evaluate these publications to determine whether they are relevant and should be cited. There is no requirement to cite these works unless the editor has indicated otherwise. | Not applicable | X |

| **Response to reviewer 1:** | | | |
| --- | --- | --- | --- |
| Comment raised | Response by authors | Location of revisions | |
| 1.This study employed a cross-sectional design with a 6-month retrospective cost trajectory analysis. This design has inherent limitations in evaluating causal relationships (e.g., the impact of cost drivers on cost trajectories). It is recommended that these limitations be explicitly stated in the discussion section, and future research consider adopting a prospective cohort design to better establish causal inferences. | Thank you for this important point. We agree that our study design is with a 6-month retrospective cost trajectory analysis limits causal inference.  We have explicitly clarified this in the Discussion. We also added that future research using quasi-experimental methods would be better suited to establish causal mechanisms underlying cost trajectories: “*In addition, our retrospective longitudinal observational study design limits causal inference. Prospective cohort designs or quasi-experimental methods (such as difference-in-differences and interrupted time series) are better suited to examine causal relationships between patient characteristics and costs.”*  Other wording that could be interpreted as implying causality rather than association has also been revised throughout the manuscript. | **Lines:**  359-362.  **Lines**:  9, 19, 30, 178, 279, 302, 328, 355. | |
| 2.The study utilized nationwide health claims data, which may be susceptible to coding bias or missing information. Please clarify the specific methods used to handle missing data, and discuss how these potential biases may have affected the identification and interpretation of cost trajectories. | Thank you for raising this important point; we agree that the handling of missing data was insufficiently described in the original manuscript. Before addressing how missing data were handled, we consider it important to first provide additional clarification on a) the data source, b) the limitations of using administrative claims data, and c) our approach to handling zero-cost observations, as these aspects were also not sufficiently described in the manuscript:  a) Data source  This study utilized administrative health claims data from Vektis, which collects all healthcare claims processed and reimbursed by health insurers and care offices under the Dutch Health Insurance Act (Zvw) and Long-Term Care Act (Wlz). These data were used to construct cost trajectories, including short-term residential care (STRC), geriatric rehabilitation (GR), district nursing care, hospital care, and long-term care. As these data capture only reimbursed healthcare expenditures, the resulting cost trajectories reflect a payer perspective.  This has been clarified in the methods section with: “*The trajectory costs reflected a payer perspective and included reimbursed healthcare expenditures across seven categories: STRC, district care, LTC at home, nursing home admission, GR, inpatient and outpatient hospital care.* ”  b) Limitations of claims data It is important to note that Vektis claims data are not primarily collected for research purposes and therefore have inherent limitations. First, they capture only reimbursed care; healthcare use that was not claimed, was rejected, or was financed outside these statutory frameworks is not observed. Second, although claims are submitted using nationally standardized coding and billing systems, variation may still arise due to differences in registration and coding practices across providers, particularly for more detailed variables such as diagnosis, despite the presence of guidelines. The logistic regression including diagnostic information prior to STRC admission (Model 2) should therefore be interpreted with caution, as diagnostic coding practices are more prone to variation between hospitals (e.g. differences in interpretation or failure to update preliminary to final diagnoses).  These limitations have now been clarified in the Discussion section by adding the following sentences: “*First, the study used administrative claims data, which are not primarily collected for research purposes and capture only reimbursed care; care that was not claimed, rejected, or financed outside statutory health insurance schemes is not included. Although national coding standards apply, variation in registration practices across providers may occur, particularly for diagnosis codes. Results from logistic regression Model 2 (including a primary categorical diagnosis variable) should therefore be interpreted with caution, given potential inter-provider differences in coding practices (e.g., variation in interpretation or updates from preliminary to final diagnoses).”*  c) Handling of zero-cost records In addition, zero-cost records were observed in the Vektis data. Within the claims data of STRC patients admitted in 2022, 275 (out of 35,142) patients had zero costs claimed during their STRC admission period. These patients were assumed not to have received STRC care and were therefore excluded from the cohort (see consort diagram in S5 Figure). For other components of the cost trajectories (GR, district nursing care, long-term care, and hospital care), zero-cost observations were interpreted as absence of healthcare use for the respective activity, given that no costs were declared.  This assumption has been clarified in the Methods section by adding the following sentence: “*Zero-cost records were interpreted as absence of healthcare use for the corresponding service.*”  **Handling of missing data: living situation & income** In Table 1, missing data were present for two patient characteristics: living situation (n = 58) and household income (n = 150). For the logistic regression analyses (Model 1 + Model 2), missing values for living situation and household income were recoded into a separate “unknown” category to retain these observations in the model. This approach preserves statistical power and avoids potential bias associated with complete-case analysis, while explicitly accounting for missingness as an informative category.  This procedure has now been clarified in the Methods section by adding the following sentence: “*Missing values for living situation and household income were recoded into a separate ‘unknown’ category to account for the possibility that missingness did not occur at random.”*  **Handling of missing data: primary diagnosis**  From the cohort used for logistic regression analyses including the categorical variable ‘primary diagnosis (Model 2, N = 7,110), patients were excluded if they did not have a GR, ED, or hospital admission prior to STRC admission, or if diagnosis data from these admissions were missing (see the consort diagram in S5 Figure).  This procedure has been clarified in the Methods section by adding the following phrase: “*or with missing primary diagnosis information were excluded (see the consort diagram in S5 Figure).* | **Lines:**  120-122.  **Lines:**  305-311.  **Lines**:  123-124.  **Lines:**  179-181.  **Lines**  184-185. | |
| 3.In group-based trajectory modelling, how are the criteria for dividing into the "high-cost", "low-cost" and "palliative care" groups determined? | Thank you for this important question. The palliative care group was defined a priori based on the financing label “STRC palliative care.” This group was not derived from the GBTM but was included separately due to its distinct clinical and cost profile. For the identification of the low- and high-cost groups, group-based trajectory modelling (GBTM) was applied. The modelling approach and selection of the number of groups are described in the Methods section ‘Identification of cost group’: (lines 147–166). In addition, Tables and Figures in S7 present the model selection criteria and diagnostics on which the selection of the two trajectory groups (low- and high-cost) was based.  We acknowledge that the distinction between trajectory groups identified through GBTM and the a priori defined group could have been clearer, particularly in the Abstract. We have therefore revised the manuscript to explicitly clarify that two groups were identified using GBTM, while the third group (palliative care) was defined a priori based on its financing label (STRC palliative care). | **Lines:**  17, 23-24, 133, 148-150, 232-235, 247, 298, 315-317. | |
| 4. The conclusion that "*Clinicians and policymakers should reconsider STRC’s role in nursing home transitions*" requires more direct empirical support. The current study only demonstrates an association between longer STRC stays and higher costs, but does not establish that STRC itself caused unnecessary nursing home transitions. It is strongly recommended to revise this statement to avoid over-interpreting causal relationships, and frame the conclusion more cautiously as a correlational finding. | Thank you for this helpful comment. We have revised the Abstract and Discussion to ensure that the implications are more closely aligned with the presented results and that the conclusions are framed more cautiously as correlational findings.  We agree that our findings do not support the conclusion that STRC itself leads to unnecessary nursing home admission. Our findings show that a substantial proportion of STRC patients were discharged to nursing homes, particularly within the high-cost group (29.8%). This raises the question for clinicians and policymakers of whether STRC is an appropriate setting for patients who are likely to require nursing home admission after discharge, given that its overarching aim is to enable patients to return home and live independently in the community.  We have reframed this point in the Discussion accordingly, so that our suggestion is more directly linked to the study finding. | **Lines**:  339-344. | |
| **Response to reviewer 2:** | | | |
| Comment raised | Response by authors | | Location of revisions |
| 1. This manuscript explores the cost trajectories of patients admitted to short-term residential care (STRC) in the Netherlands. It performs Group-Based Trajectory Modeling (GBTM) on Dutch claims data. This raises expectations about distinguishing groups based on differences in healthcare utilization patterns. The topic of the manuscript is relevant and the authors uses a large dataset concerning the entire relevant patient population. | Thank you for this positive and encouraging assessment of our work. We appreciate your recognition of the relevance of the topic and the use of nationwide claims data covering the full target population. | | X |
| 2. My main feedback to improve this manuscript is that conclusions are drawn that are not supported by the results. The authors may consider framing these suggestions more cautiously or linking them more directly to the study findings.   a) “*Clinicians and policymakers should reconsider STRC’s role in nursing home transitions and improve transitional care*”. High costs may indicate the importance of STRC, but they do not necessarily mean that STRC is being used incorrectly. b) Also, the statement “*Our findings suggest that STRC does not fully meet its objective of enabling patients to return home and live independently*” may require an explicit evaluation or comparison in some form. c) Furthermore, the policy implications (e.g., refined admission criteria and triage tools) appear speculative given the presented results. | Thank you for this helpful comment. We have revised the manuscript to better align the implications presented in the Abstract, Discussion and Conclusion with the results of our study, ensuring that our interpretations are more closely grounded in the findings.  a) We agree that high costs do not necessarily mean that STRC is being used incorrectly. However, our findings show that a substantial proportion of STRC patients was discharged to nursing homes, particularly within the high-cost group (29.8%). This raises the question for clinicians and policymakers of whether STRC is an appropriate setting for patients who are likely to require nursing home admission after discharge, given that its overarching aim is to enable patients to return home and live independently in the community. We have reframed this point in the Discussion accordingly, so that our suggestion is more directly linked to the study finding.  b) We agree that the statement “*Our findings suggest that STRC does not fully meet its objective of enabling patients to return home and live independently*” was not sufficiently supported by our results. We have removed this statement and related references throughout the manuscript. Thank you for this critical observation.  c) We also agree that the policy implications regarding refined admission criteria, triage tools, and structured multidisciplinary assessment at intake were too speculative given the available evidence, and we have therefore removed these statements. | | **Lines**:  339-344.  **Lines**:  327, 371-374.  **Line:**  354. |
| 3. The manuscript reports three cost groups; however, only two are based on the GBTM, while the other one is defined by the researchers themselves (i.e., not a latent group). This is not clear throughout the text, including in the abstract, even though it is particularly relevant there. | Thank you for this important question. The palliative care group was defined a priori based on their funding category ‘STRC palliative care’. This group was not derived from the GBTM but was included separately due to its distinct clinical and cost profile.  We acknowledge that the distinction between trajectory groups identified through GBTM and the a priori defined group could have been clearer, particularly in the Abstract. We have therefore revised the manuscript to explicitly clarify that two groups were identified using GBTM, while the third group (palliative care) was defined a priori based on its funding category (STRC palliative care). | | **Lines:**  17, 23-24, 133, 148-150, 232-235, 247, 298, 315-317. |
| 4. The term “cost drivers” is used, which presupposes causality, whereas the so-called drivers themselves are part of the costs. This therefore requires a different, less causal formulation. | Thank you for this important point. We agree that our study design with a 6-month retrospective cost trajectory analysis limits causal inference.  Wording that could be interpreted as implying causality rather than association has also been revised throughout the manuscript.  We have also changes our wording in the discussion section and added that future research using quasi-experimental methods would be better suited to establish causal mechanisms underlying cost trajectories: “*Our logistic models, assessing associations between patient characteristics and high-cost group membership, showed low explanatory power, with most variance remaining unexplained. This suggests unobserved heterogeneity (e.g. somatic, functional, cognitive, and social vulnerability) that administrative claims data do not capture. For future risk-stratification in STRC, we recommend linking claims data with clinical or survey-based datasets. In addition, our retrospective longitudinal observational study design limits causal inference. Prospective cohort designs or quasi-experimental methods (such as difference-in-differences and interrupted time series) are better suited to examine causal relationships between patient characteristics and costs.”* | | **Lines**:  9, 19, 30, 178, 279, 302, 328, 355.  **Lines:**  355-362. |
| 5. In the manuscript the study is labelled as a cross-sectional study. However, since the claims data covers not just one moment in time but multiple months, this may not be the correct description of the study type. | Thank you for this important observation. We agree that describing the study as cross-sectional is not accurate, given that the claims data cover multiple time points and were used to construct cost trajectories over time. We have therefore revised the manuscript to better reflect the study design. Specifically, we now describe the study as a retrospective longitudinal observational study using administrative claims data. This change has been implemented consistently throughout the manuscript.  We have also removed references to the cross-sectional nature of the primary outcome (cost trajectories across STRC, district care, LTC at home, nursing home admission, GR, and inpatient and outpatient hospital care) throughout the manuscript to avoid confusion between ‘cross-sectional’ and ‘cross-sectoral’. | | **Lines:**  13, 96, 359-360.  **Lines:**  297, 366. |
| 6. For my understanding, why was a censored normal distribution considered appropriate for cost data? Healthcare costs are typically right-skewed and are often modeled using a gamma distribution. | Thank you for this thoughtful comment. We agree that healthcare cost data are typically right-skewed and are often modelled using gamma-based approaches in standard regression settings. However, when implementing GBTM in Stata, only Normal, Censored normal, Poisson, Zero-inflated, and Logistic distributions are supported. The censored normal was therefore the most suitable option within this modelling framework, as it accommodates the non-negative nature of cost data (unlike the Normal distribution), while the alternatives were not appropriate given that our outcome did not represent count (Poisson), excess-zero count (zero-inflated), or binary (logistic) data.  We would also note two features of our specification that help address the skewness concern. First, we modelled *cumulative* rather than monthly costs across three phases (T1 pre-admission, T3 admission, T5 post-discharge). Cumulative costs are less skewed than individual monthly observations because aggregation dampens extreme values in any single month. Second, to verify that the resulting trajectory groups were not artefacts of the distributional choice, we conducted a robustness check in the subgroup of patients surviving the full six-month period (n = 11,091; S8 Tables and figures). This subgroup analysis yielded similar latent cost trajectory patterns, supporting the stability of the identified groups.  We acknowledge that a gamma-based specification would in principle be preferable for strictly right-skewed cost data, and this is a genuine methodological limitation inherent to GBTM implementations in Stata. We have added a brief note to the Methods to make this reasoning transparent for readers. | | **Lines:**  154-158. |
| 7. Why should overlap with 2021 be avoided? (line 106) | Thank you for this question. We avoided overlap with 2021 because healthcare utilization during that period was still substantially affected by COVID-19-related disruptions, such as delays in care, staffing shortages, and changes in admission criteria. Including observations overlapping with 2021 could therefore have introduced bias and reduced comparability of cost trajectories. This assumption has been clarified in the Methods section by adding the following sentence: *“.. when healthcare utilization was substantially affected by COVID-19 disruptions…*” | | **Lines:**  111-113 |
| 8. I hope these comments are helpful to the authors in further improving the manuscript. | Thank you for these constructive and helpful comments; we appreciate the time and effort you have taken to review our manuscript and believe your suggestions have strengthened the paper. | | X |
